# Evolution of higher torque in *Campylobacter-*type bacterial flagellar motors

**DOI:** 10.1038/s41598-017-18115-1

**Published:** 2018-01-08

**Authors:** Bonnie Chaban, Izaak Coleman, Morgan Beeby

**Affiliations:** 1 Department of Life Sciences, Imperial College of London, London, SW7 2AZ UK; 2 Present Address: Faculty of Science, Health, Education and Engineering, University of the Sunshine Coast, 90 Sippy Downs Drive, Sippy Downs, 4556 QLD Australia

## Abstract

Understanding the evolution of molecular machines underpins our understanding of the development of life on earth. A well-studied case are bacterial flagellar motors that spin helical propellers for bacterial motility. Diverse motors produce different torques, but how this diversity evolved remains unknown. To gain insights into evolution of the high-torque ε-proteobacterial motor exemplified by the *Campylobacter jejuni* motor, we inferred ancestral states by combining phylogenetics, electron cryotomography, and motility assays to characterize motors from *Wolinella succinogenes*, *Arcobacter butzleri* and *Bdellovibrio bacteriovorus*. Observation of ~12 stator complexes in many proteobacteria, yet ~17 in ε-proteobacteria suggest a “quantum leap” evolutionary event. *Campylobacter-*type motors have high stator occupancy in wider rings of additional stator complexes that are scaffolded by large proteinaceous periplasmic rings. We propose a model for motor evolution wherein independent inner- and outer-membrane structures fused to form a scaffold for additional stator complexes. Significantly, inner- and outer-membrane associated structures have evolved independently multiple times, suggesting that evolution of such structures is facile and poised the ε-proteobacteria to fuse them to form the high-torque *Campylobacter-*type motor.

## Introduction

How molecular machines evolve and develop in complexity is a fundamental question for molecular biology. A central challenge to understanding protein evolution, however, is that while the fossil record can provide clues of intermediary ancestral forms for larger eukaryotic organisms, at the molecular level, we are limited to inferring ancestral states by observation of contemporary diversity.

One of the best-studied molecular machines is the bacterial flagellar motor. The flagellar motor is a periplasm-spanning rotary motor that uses proton flux to spin a multi-micron filament that coils to act as a propeller for bacterial motility. The complete flagellar structure measures approximately 50 nm wide, 10 µm long, and weighs approximately 1 billion Da^1–3^. Torque is generated by proton flux through inner membrane stator complexes, MotA_4_B_2_, that exert force on the cytoplasmic C-ring; C-ring torque is transmitted by a rigid rod across the periplasm to the extracellular propeller. Other components beside these core motor structures include a dedicated type III secretion system (T3SS) export apparatus that recruits, unfolds, and exports axial flagellar components; the inner membrane MS-ring that houses the the T3SS, and the P- and L-rings that act as bushings and portals through the peptidoglycan and outer membrane, respectively. These core components are conserved across all bacterial genera that possess flagellar systems, with the motors found in *Escherichia coli* and *Salmonella enterica* exemplifying this structure^2^. These basic motors have been extensively studied and biophysical parameters like the motor torque and overall bacterial cell swimming speed are known^4,5^.

Recently described variation in motor structure and mechanical output offer the opportunity to infer events and selective benefits in their evolution. The flagellar motors from other bacteria, such as *Vibrio* species, *Helicobacter pylori* and *Campylobacter jejuni*, are known to generate higher torque and have faster swimming speeds in high-viscosity environments^6–8^. Structural studies have revealed that these bacteria have continued to evolve and adapt motor structure by incorporating additional components^2,8^. The fact that many bacterial genera contain slightly different, yet related, versions of the flagellar motor creates a unique opportunity to use it as a model system for investigating the evolution of molecular machines.

How the high-torque ε-proteobacterial flagellar motor evolved is particularly interesting as it is one of the most structurally complex motors yet discovered. Electron cryo-tomography and sub-tomogram averaging studies of the model ε-proteobacterial motor from *C. jejuni* have revealed that large structural elaborations to the motor are responsible for its high torque. A large scaffold structure named the ‘disk complex’ composed of three periplasmic structures facilitates incorporation of a wider ring of stator complexes, increasing the lever contact point at which the stator complexes contact the C-ring, and results in exertion of greater leverage for rotation of the flagellar filament^8^. In turn, this wider ring facilitates incorporation of 17 stator complexes as compared to the ~11 in *S. enterica* and *E. coli* motors^9–11^, further increasing torque. Furthermore, unlike the load-dependent incorporation of *S. enterica* stator complexes, which renders stator complexes invisible in *in situ* subtomogram average structures under the low load conditions used for sample preparation, the *C. jejuni* stator complexes are permanently incorporated, or at least present at very high occupancy, and are clearly resolved in subtomogram average structures of the *in situ* motor. Such high occupancy likely yet further boosts torque in lower viscosity conditions that may predispose *C. jejuni* for motility immediately upon transition to a high-viscosity environment. *C. jejuni’*s motor torque and likely continuous high energy consumption are consistent with its habitat of the animal gut, where nutrient availability is high, but the environment is highly viscous.

The *Campylobacter-*type motor’s disk complex is formed of at least four additional structural proteins not present in *S. enterica* or *E. coli* ^8,12^. The basal disk, located just below the outer membrane and formed from FlgP, may support the outer membrane during motor rotation. Assembly of the basal disk is required for assembly of the medial ring, formed of PflA and possibly FlgQ. Last to assemble is the inner membrane-associated proximal ring formed from PflB. The proximal ring has become essential for the incorporation of the stator complexes into the *Campylobacter-*type motor^8^.

How did the *Campylobacter-*type motor evolve from a simpler ancestral motor? During our previous work, we discovered that each accessory protein is essential, posing a conundrum: how could proteins have been added stepwise to form this (naively “irreducibly complex”) motor? To identify a possible incremental evolutionary pathway, we determined a phylogeny of ε-proteobacterial and related motors, used different accessory protein occurrences to identify and determine structures of the likely descendants of evolutionarily intermediate motors found in *Wolinella succinogenes*, *Arcobacter butzleri*, and *Bdellovibrio bacteriovorus. W. succinogenes* is a cattle rumen commensal^12,13^ while *A. butzleri* is a human gastrointestinal pathogen^12,14^, similar environments to *C. jejuni*. The more distantly related *B. bacteriovorus* can be found in a number of habitats including sewage and the human gut^15^. Subsequent to imaging the motor structures we related their structure to mechanical output using swimming speed assays. Our results enable us to propose a model for how the *Campylobacter-*type motor evolved by inferring a possible scenario for the order of protein recruitment events, and the selective benefits at each step.

## Results

### Motor phylogeny reveals the scope of ε-proteobacterial flagellar diversity

**Figure 1:**
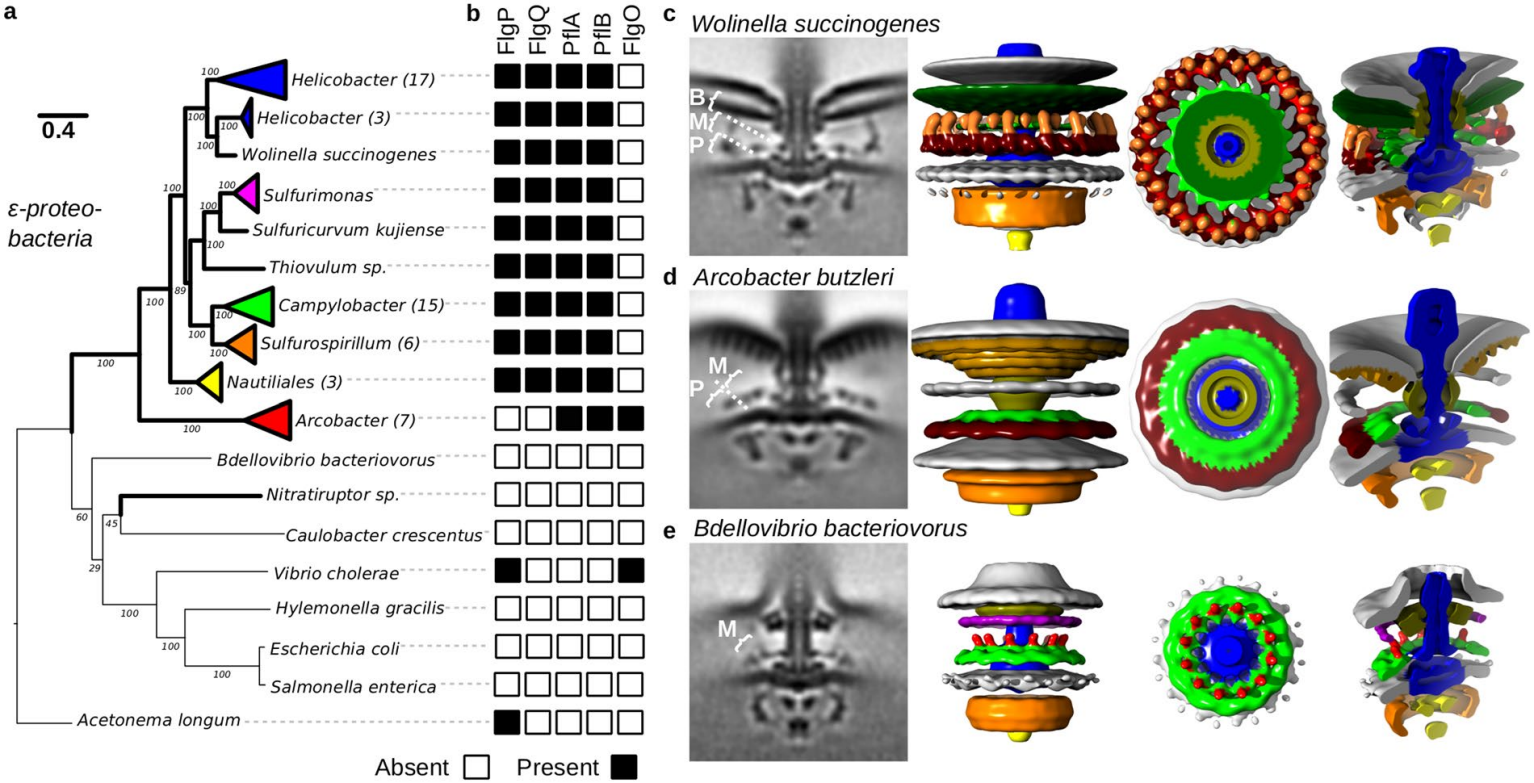
Diverse flagellar motors are suggestive of an evolutionary pathway to the high-torque ε-proteobacterial flagellar motor. (**a**) Phylogenies of 11 core flagellar motor proteins with focus on ε-proteobacteria. Parentheses after genera names on the tree indicate the number of unique strains represented by the collapsed branch. The extended and bootstrapped version of this tree is presented in Supplementary Fig. S1. (**b**) Presence (black square) and absence (white square) of accessory proteins FlgP, FlgQ, PflA, PflB, and FlgO found in the corresponding genome sequences. (**c**–**e**) Subtomogram averages of motors investigated; l–r: 100 × 100 nm slice through the centre of the subtomogram average, isosurface, top-down view of the stator plane, tilted view of cross-section: (**c**) *Wolinella succinogenes*, (**d**) *Arcobacter butzleri*, and (**e**) *Bdellovibrio bacteriovorus*. Pale green represents possible PflA density, dark red possible PflB, and bright red putative stator complexes. “B”, “M”, and “P” labels depict Basal disk, Medial ring, and Proximal rings respectively, as previously proposed for *C. jejuni* ^8^ (see also Fig. 2). Unsymmetrized structures and FSC curves for each flagellar motor can be found in Supplementary Fig. S4.

To better understand ε-proteobacterial motor evolution, we sought to first determine the phylogeny of the core motor by concatenation of component protein sequences (Fig. 1a). Strains were chosen for analysis to represent broad coverage across fully sequenced ε-proteobacterial genomes. Sequences of 11 core motor proteins (C-ring proteins FliG, FliM, and FliN, T3SS proteins FlhB, FliI, FliP, and FliR, proximal rod proteins FlgB, FlgC, and FliE, and MS-ring protein FliF; Supplementary Table S1) from the core ε-proteobacterial flagellar motor were identified in each species, concatenated, and used to build a maximum likelihood motor phylogeny. Phylogenies of all 11 individual proteins were congruent.

To relate motor phylogeny to species evolution, an organism phylogeny was determined using concatenated ribosomal protein sequences (Supplementary Table S2). Supplementary Figs S1 and S2 contain the complete, bootstrapped flagellar motor and ribosomal protein trees, respectively. Unexpectedly, comparison of organism and motor phylogenies showed that while all *Arcobacter* motors branch from the root of the ε-proteobacterial motor clade (Supplementary Fig. S3), the ribosomal protein-derived organism phylogeny indicates that all *Arcobacter* species diverge from within the ε-proteobacteria, adjacent to the *Campylobacteriales*. Intriguingly, the phylogenies of four proteins (omitted from the concatenated motor phylogeny), L-ring protein FlgH, P-ring protein FlgI, and T3SS proteins FlhA and FliQ, more closely matched *Arcobacter*’s organism phylogeny than the core proteins used to determine motor phylogeny. The *flgH*, *flgI*, *flhA* and *fliQ* genes were distributed across *Arcobacter* flagellar gene clusters and furthermore were the sole copies of each gene in their respective genomes, indicating that *Arcobacter* motor must assemble using these proteins. Both the core *Arcobacter* motor phylogeny, and FlgH, FlgI, FlhA and FliQ, however, branch from the within the ε-proteobacterial motor tree. A second incongruency was noted in the branching position of the deep-sea, hydrothermal vent inhabiting ε-proteobacterium *Nitratiruptor*, which branches from outside the ε-proteobacterial motor clade, suggesting an ancestral horizontal transfer of a non-ε-proteobacterial entire flagellar motor system to an ancestor of *Nitratiruptor*.

### Presence of known accessory proteins suggests intermediary motor states

To identify contemporary descendants of evolutionarily intermediate motors to the *Campylobacter-*type motor, we charted the occurrence patterns of previously identified flagellar motor accessory proteins. We searched for accessory proteins FlgP, FlgQ, FlgO, PflA, PflB, FlgT, MotX and MotY from the previously characterized motors of *C. jejuni* and *Vibrio* species^2,8^. No homologs of FlgT, MotX, or MotY were identified in ε-proteobacteria and were therefore omitted from further study.

Sequence searches for flagellar motor accessory proteins revealed two types of accessory protein presence within the ε-proteobacteria (Fig. 1b): the canonical *Campylobacter*-type containing FlgP, FlgQ, PflA and PflB and *Arcobacter*-type motors containing PflA and PflB and, unexpectedly, a homolog of the *Vibrio* flagellar protein FlgO. Neither of these patterns of accessory protein occurrence were detected outside of the ε-proteobacteria. Although we did not identify any of the accessory proteins in the δ-proteobacteria by homology searches, we noted a protein encoded within a *B. bacteriovorus* flagellar operon that echoes the domain architecture of PflA with an N-terminal signal sequence and transmembrane helix followed by a very long (~1000 amino acid) stretch of TPR motifs (WP_011165745.1). Because this protein is encoded in a flagellar operon, we retained *B. bacteriovorus* for further analysis.

### The *Wolinella succinogenes* motor is intermediate to *Campylobacter* and *Helicobacter* motors

Although examples of the *Campylobacter-*type motor accessory protein configuration exist with the *C. jejuni*, *H. hepaticus*, and *H. pylori* motor structures, the breadth and diversity of the *Campylobacterales* order prompted acquisition of an additional example. A subtomogram average of the *W. succinogenes* flagellar motor was determined from 154 motor particles (Fig. 1c) (Supplementary Fig. S4A).

The *W. succinogenes* motor was very similar to the *C. jejuni* motor. Both motors have 17-fold symmetry in their proximal and medial ring structures according to nomenclature previously introduced^8^. Densities corresponding to the previous experimental determination of the identity of MotB in the closely related *C. jejuni* motor^8^ were clearly visible, indicating that *W. succinogenes* also has 17 stator complexes. The appearance of the basal disk structure (named according to previously introduced nomenclature^8^) in the sub-tomogram average is consistent with previous studies of the basal disk from *W. succinogenes* by purification and negative stain^16^. These studies suggested the disk is formed as an Archimedean spiral, which would appear as a solid density if alignment is driven by the prominent 17-fold symmetry of the proximal and medial rings^16^ (Fig. 1c). The only major point of difference between the *C. jejuni* and *W. succinogenes* structures is in the area of the *C. jejuni* medial ring, which in *C. jejuni* extends parallel to the proximal ring and basal disk; in *W. succinogenes* a perpendicular structure bridges between the proximal ring and basal disk further from the central rod (Fig. 1c). The FlgQ homologs from the two species are quite distinct and these sequence differences may contribute to the structural differences.

### The *Arcobacter butzleri* motor suggests the structure of the common ancestor of ε-proteobacterial motors

**Figure 2:**
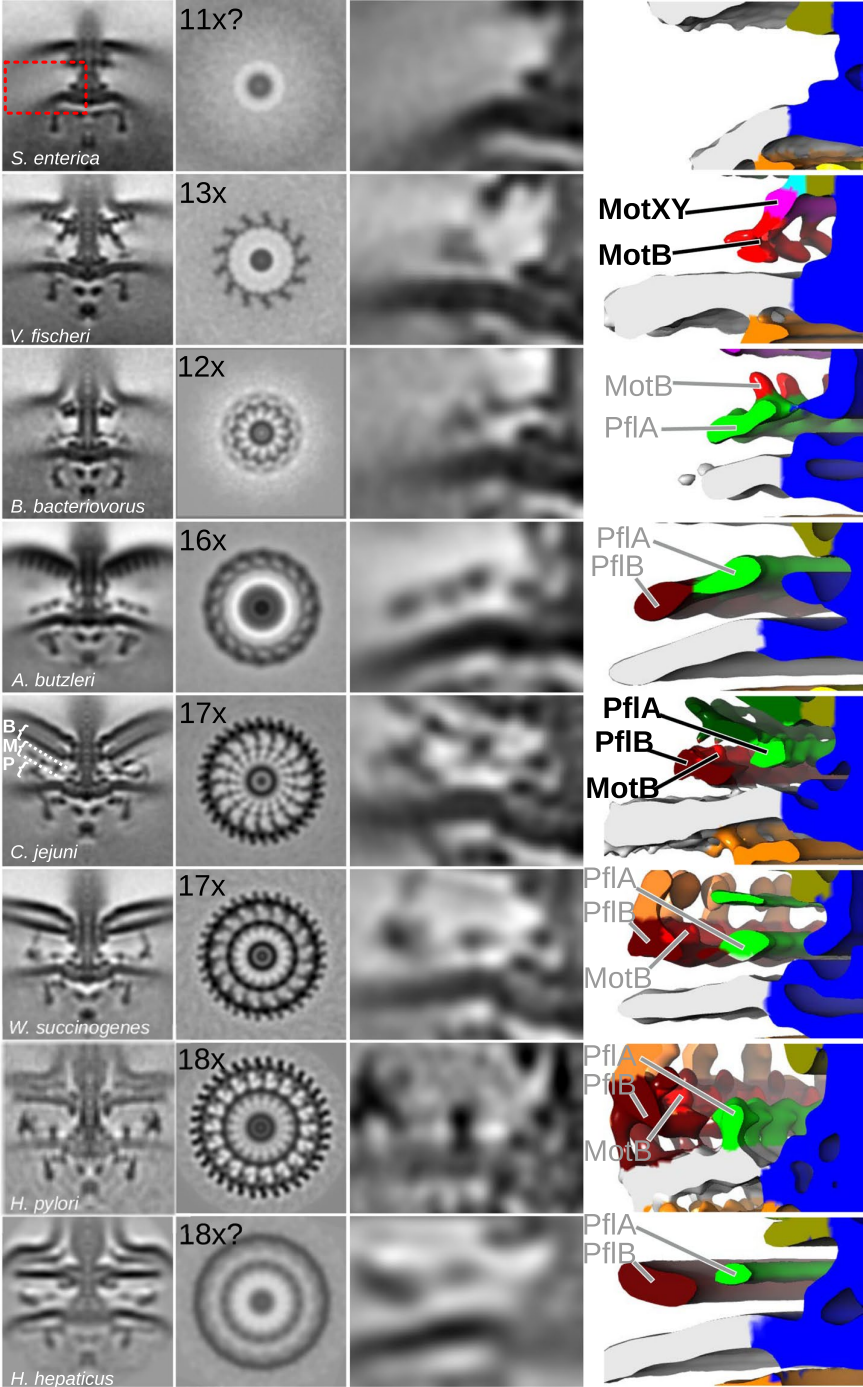
Broader context of bacterial flagellar motor diversity enables inference of ancestral states. Subtomogram averages (as profile and slice through proximal and medial ring structures) of relevant bacterial flagellar motors from this and previous studies. Panels, l–r: 100 × 100 nm slice through the centre of the motor; 100 × 100 nm cross-section through the motor at the stator plane; close-up of periplasmic and inner membrane region highlighted by red box for *S. enterica* motor; isosurface rendering of close-up of periplasmic and inner membrane region. Isosurface labels in black denote proteins whose locations have been experimentally determined; labels in grey represent protein locations inferred from experimental determinations in related organisms. Motor species, from top to bottom: *S. enterica* ^8^, *V. fischeri* ^8^, *B. bacteriovorus* (this study), *A. butzleri* (this study), *C. jejuni* ^8^, *W. succinogenes* (this study), *H. pylori* ^17^, and *H. hepaticus* ^2^. “B”, “M”, and “P” labels depict Basal disk, Medial ring, and Proximal rings respectively for the *C. jejuni* motor.

The structure of the *Arcobacter*-type motor was determined using subtomogram averaging of 478 *A. butzleri* flagellar motors (Fig. 1d) (Supplementary Fig. S4B). Given the shared presence of the flagellar accessory proteins PflA and PflB in the genomes of species used to image the *Arcobacter*-type and *Campylobacter*-type motors, the proximal ring from *A. butzleri* was, as expected, similar to the proximal ring in *C. jejuni* and *W. succinogenes* (Fig. 1c). Unlike the 17-fold symmetry observed in the proximal rings of *C. jejuni* and *W. succinogenes*, the proximal ring from *A. butzleri* was 16-fold symmetric. *A. butzleri*’s proximal ring, however, lacked stator complex densities. The most plausible interpretation of this lack of density is that *A. butzleri* stator complexes are dynamic like *S. enterica* and *E. coli* stator complexes, and therefore at low occupancy under the low load imaging conditions (Fig. 2).

Consistent with *Arcobacter* species lacking homologs of FlgP or FlgQ, its motor lacked the corresponding basal disk structure. The *A. butzleri* motor did, however, contain an outer membrane-associated disk structure distinct to the *C. jejuni* and *W. succinogenes* basal disk. This analogous disk was tightly associated with the outer membrane, in contrast to the separation from the outer membrane seen with the FlgP-derived basal disk (Fig. 1d). Unlike the FlgP basal disk’s Archimedean spiral, the *A. butzleri* basal disk is composed of 5–6 concentric rings visible as distinct dots in the subtomogram averages (Fig. 1d). Notably, this is strikingly similar to the FlgO-containing basal disks found in γ-proteobacteria like *Vibrio fischeri* (Fig. 2)^8^. While the *V. fischeri* basal disk is hypothesized to contain FlgO and FlgP, it is likely that the FlgO homolog is part of the *A. butzleri* disk structure.

No structures were observed linking the inner- and outer-membrane-associated structures in the *A. butzleri* motor. Based on its phylogenetic context, accessory protein occurrences, and structure of the *A. butzleri* motor, it is likely that the last common ancestor of the *Arcobacter-*type and the *Campylobacter-*type motors had not yet incorporated a FlgP basal disk.

### Structure of the *Bdellovibrio bacteriovorus* motor suggests a precursor state

Although δ-proteobacteria are closely related to ε-proteobacteria and promise insights into ε-proteobacterial motor evolution, no δ-proteobacterial motor structures have been determined to-date. The δ-proteobacterium *Bdellovibrio bacteriovorus* branches between the ε-proteobacteria clade and the ε-proteobacterium *Nitratiruptor*, and furthermore encoded a protein in a flagellar operon whose domain architecture resembled the ε-proteobacterial PflA, despite lacking significant sequence similarity. Toward inferring the structure of the precursor of the ε-proteobacterial motors, we determined the structure of the *B. bacteriovorus* motor by subtomogram averaging 206 motor particles (Fig. 1e) (Supplementary Fig. S4C).

The *B. bacteriovorus* motor structure was considerably smaller than any of the ε-proteobacterial motors (Fig. 1e), but featured a small ring of unknown protein composition associated with the inner membrane. Given the presence of the PflA-like protein encoded in a flagellar operon in *B. bacteriovorus*, and the correspondence of this ring with the *C. jejuni* PflA ring we speculate that this ring is composed of the *B. bacteriovorus* protein WP_011165745.1. If this is the case, *B. bacteriovorus* may represent a descendant of the first motor to incorporate a PflA homolog prior to incorporation of any of the other accessory proteins. Alternatively this ring may be a convergent evolution of a distinct inner membrane-associated ring.

The stator complexes are expected to be located in the area of the small ring. The ring was 12-fold symmetric suggesting that it interacts with up to 12 stator complexes. Indeed, we observed a periplasmic density projecting ~10 nm into the periplasm directly above the outer lobe of the C-ring, as previously observed for the stator complexes in *C. jejuni*, *V. fischeri*, spirochaetes, and *H. gracilis* ^2^.

In addition to the inner membrane-associated ring, the *B. bacteriovorus* P- and L-rings are enlarged relative to the *S. enterica* structures, interacting with the outer membrane at the point of transition to the sheath, although the identity of the protein(s) that enlarge these structures are unknown. These features distinguish the *B. bacteriovorus* motor from ε-proteobacterial flagellar motors and confirms the branching pattern seen in the motor phylogeny (Fig. 1a)^2,8,9^.

### Comparison of motor structures enables interpretation of motor phylogeny

To interpret our data, we compared our new structures to previously reported structures (Fig. 2). Eight motors were compared: *S. enterica* ^8^, *V. fischeri* ^8^, *B. bacteriovorus* (this study), *A. butzleri* (this study), *C. jejuni* ^8^, *W. succinogenes* (this study), *Helicobacter pylori* ^17^, and *Helicobacter hepaticus* ^2^. By comparing established locations of MotB, PflA, PflB, and FlgP from previous studies, comparison of structures in light of phylogeny and accessory protein occurrences enables inference of protein identities in other motors.

The phylogenetic profiles of PflA and PflB, together with previous work confirming their locations in the *C. jejuni* motor matched corresponding densities in diverse bacteria. The phylogenetic profile of PflA was consistent with an additional periplasmic density immediately adjacent to the MS-ring in PflA-containing motors from *A. butzleri*, *C. jejuni*, *W. succinogenes*, and the two *Helicobacter* species. This density has previously been shown to be PflA in *C. jejuni* ^8^. Furthermore, the *B. bacteriovorus* motor features a similar density, consistent with the presence of a speculative distant PflA homolog in its flagellar operon. We conclude that this density (marked as a green density in Fig. 2) corresponds to PflA in these organisms. As with PflA, the phylogenetic profile of PflB matched a second, wider, periplasmic ring around PflA in *A. butzleri*, *C. jejuni*, *W. succinogenes*, and the two *Helicobacter* species. As with PflA, this density has previously been confirmed to be PflB in *C. jejuni* ^8^ (marked as a dark red density in Fig. 2). We conclude that these two proteins play conserved roles in diverse bacteria, forming rings at similar locations in the periplasm.

Structures were further compared to determine the location, number, and dynamics of stator complexes. Although the *B. bacteriovorus* structure was of insufficient resolution to discern the motor’s lever contact point, location of the outer lobe of the C-ring at a 20 nm radius, and position of 12 putative stator complexes at the same radius suggests a similar lever contact point to the estimated *S. enterica* lever contact point of 20 nm^8^. Unlike *B. bacteriovorus*, we could not resolve periplasmic stator complex densities in *A. butzleri*. Consistent with this, we could not resolve a cytoplasmic density at the lever contact point connecting stator complexes with the outer lobe of the C-ring. Nevertheless, the outer lobe of the C-ring is positioned at a radius of 27 nm from the axis of rotation, similar to the contact lever points of *C. jejuni* and *W. succinogenes* of 26.5 nm and 27 nm, respectively^8^. Whether *A. butzleri* stator complexes behave similar to the enteric stator complexes – i.e., engage as a function of motor load – remains unknown. The *W. succinogenes* motor structure was very similar to *C. jejuni*, with 17 densities that matched the location of the 17 stator complexes in *C. jejuni* ^8^ (marked as bright red density in Figs 1 and 2), with a lever contact point positioned at a radius of 27 nm from the axis of rotation, comparable to the *C. jejuni* contact lever point radius of 26.5 nm^8^. Finally, the two *Helicobacter* structures were very similar to *W. succinogenes*. Both motors share the perpendicular medial ring (termed ‘the cage’ in *H. pylori*). Significantly, however, the *H. pylori* proximal ring has 18-fold symmetry that will support up to 18 stator complexes in comparison to the 17-fold symmetry of *W. succinogenes* and *C. jejuni*. The previously determined *H. hepaticus* structure is of insufficient resolution to determine the number of stator complexes, but the radius of the stator ring matches the radius of the 18-fold symmetric *H. pylori* stator ring.

### Bacterial swimming ability is consistent with structural predictions of motor output

Following from work showing that motor structure is a good predictor of motor torque^8^, our structures indicate that motor torque increases as motors develop large scaffolds that increase the radius of the contact lever point, increase the maximum number of stator complexes, and increase the occupancy of individual stator complexes. To evaluate this in the physiological context of bacterial swimming, we sought to assess the swimming ability of different species in different environments

**Figure 3:**
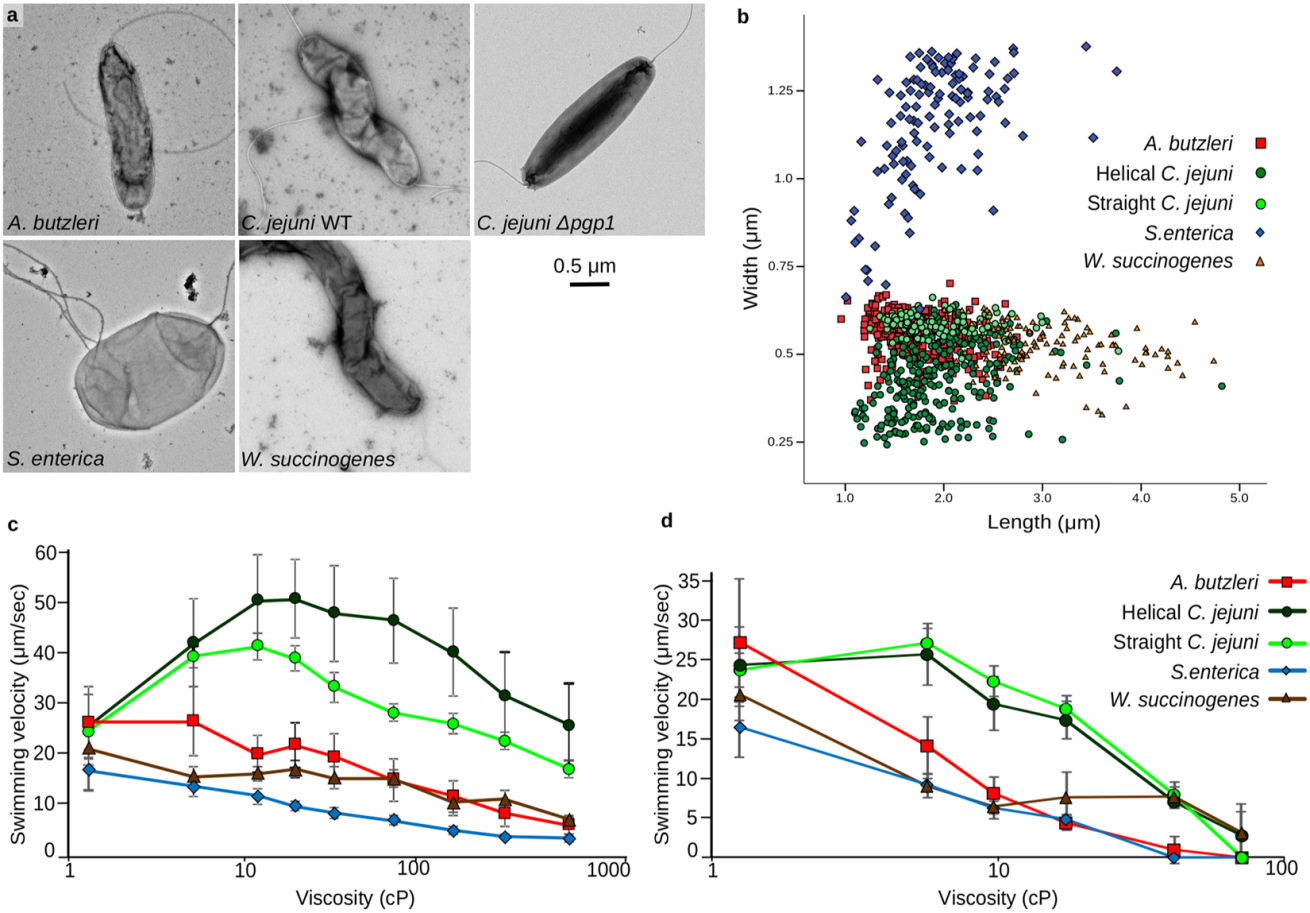
Determination of bacterial swimming speed in methylcellulose and Ficoll viscosity solutions. (**a**) Negative stained images of the bacterial strains tested; *A. butzleri*, *C. jejuni* wild type, *C. jejuni* Δ*pgp1* mutant (straight cell type), *S. enterica*, and *W. succinogenes*. (**b**) Cell size measurements of individual cells from each type. Swimming velocities of strains in methylcellulose (**c**) or Ficoll (**d**) solutions of increasing viscosity, reported as the average swimming speeds of two independent cultures, recorded in triplicate, containing the swimming speeds of >600 individual cells and reported in µm/sec ± one standard deviation.

To evaluate the impact of differences in cell lengths and widths of *C. jejuni*, *W. succinogenes*, *A. butzleri* and *S. enterica* on motility (Fig. 3a), we first quantified the dimensions of all four species. The length and width measurements for each cell measured is shown in Fig. 3b, with the average *C. jejuni* cell being 1.93 ± 0.47 µm long and 0.43 ± 0.11 µm wide, the average *A. butzleri* cell being 1.74 ± 0.35 µm long and 0.57 ± 0.05 µm wide, the average *W. succinogenes* cell being 3.08 ± 0.62 µm long and 0.52 ± 0.07 µm wide, and the average *S. enterica* cell being 1.92 ± 0.49 µm long and 1.15 ± 0.18 µm wide. By one-way ANOVA, both the lengths (F(4,783) = 175.419, p < 0.001) and widths (F(4,783) = 1124.4, p < 0.001) of the different species are significantly different. Cell shapes between species also differed (Fig. 3a). *S. enterica* is a small rod, with a generally straight body, *A. butzleri* is a straight to slightly crescent-shaped rod, and both *C. jejuni* and *W. succinogenes* are helical, with an average of 4 ± 1 and 3 ± 1 turns per cell, respectively. Given that cell shape can impact swimming speed performance in some viscous environments, we obtained a *C. jejuni* Δ*pgp1* mutant that produces straight cells for comparison^18^. This mutant strain still possesses a wild type *C. jejuni* flagellar motor and resulted in *C. jejuni* “straight” cells with a similar average length to wild-type *C. jejuni* (1.92 ± 0.42 µm) but slightly wider (0.59 ± 0.03 µm).

To characterize motility and differentiate the contributions of cell size and shape on swimming speed measurements, we compared bacterial motility in both methylcellulose and Ficoll viscous solutions. Methylcellulose is a long, branched polymer that forms a structured matrix in solution whose addition has previously been shown to be advantageous for motility of helical bacteria^19^. In this situation, narrower and more curved or helical cell shapes would be expected be able to exert greater thrust through a ‘corkscrewing’ motion of their cell bodies through the matrix. Ficoll, meanwhile, is a spherical polymer that forms a more unstructured solution^19^, where cell shape has less impact. Swimming velocity measurements in increasing viscosities were obtained for the five test cultures in both methylcellulose (Fig. 3c) and Ficoll (Fig. 3d) solutions. The effect of cell shape was evident by comparing wild type (helical) *C. jejuni* and Δ*pgp1* (straight) *C. jejuni* between the two solutions. The methylcellulose environment showed wild type *C. jejuni* to have an increased swimming speed at all viscosities tested compared to its straight-mutant counterpart (Fig. 3c). Conversely, no difference in the swimming performance between the *C. jejuni* strains was detected in the Ficoll solution (Fig. 3d). We conclude that comparisons of swimming performance between species is best undertaken in Ficoll solutions that do not provide a motility advantage to different cell shapes.

*S. enterica* has the simplest motor with no disk complex and a predicted 11 dynamic stator complex capacity. Despite assembly of ~5 motors per cell, *S. enterica* had the slowest swimming speed and was incapable of motility at higher viscosities (Fig. 3d). *A. butzleri*, with its predicted 16 stator complex capacity and dynamic stator complex occupancy, swam faster than *S. enterica* at low viscosities, but slowed to *S. enterica* speeds and became trapped by higher viscosities (Fig. 3d). Both *C. jejuni* and *W. succinogenes*, with complete disk complexes for their 17 stator complex capacity and with high stator complex occupancy continue swimming at viscosities that immobilize *S. enterica* and *A. butzleri* (Fig. 3d). Both the *C. jejuni* wild type and straight-mutant strains had superior swimming performance at all viscosities tested as expected given their wider rings of additional stator complexes. *W. succinogenes* had a relatively slower swimming speed in the lower viscosity environments that we attribute to increased frictional resistance on its longer and wider cell body. Furthermore, results may also reflect the fact that the bacteria investigated are variously aerobic, microaerophilic, and anaerobic. Indeed, *W. succinogenes’* anaerobic lifestyle may contribute to its lower swimming speeds in addition to, or as well as, drag from its longer, wider body.

These swim assays support the hypothesis that increased stator complex capacity and additional motor structural supports result in higher torque to improve cell swimming performance in viscous environments.

## Discussion

In this study we combined phylogenetic, structural, and phenotypic studies to understand by inference possible evolutionary pathways to high torque in the *Campylobacter-*type ε-proteobacterial motors. By comparing motor structures and swimming ability from different species, it is clear that the evolution of this machine is more complex than we first proposed^8^. Our observations, however, provide sufficient constraints to propose a model for its evolution, and suggest general evolutionary principles. Each intermediate motor structure fills a “missing link” in motor diversity, with each exhibiting advantages over simpler relatives, explaining continued retention.

**Figure 4:**
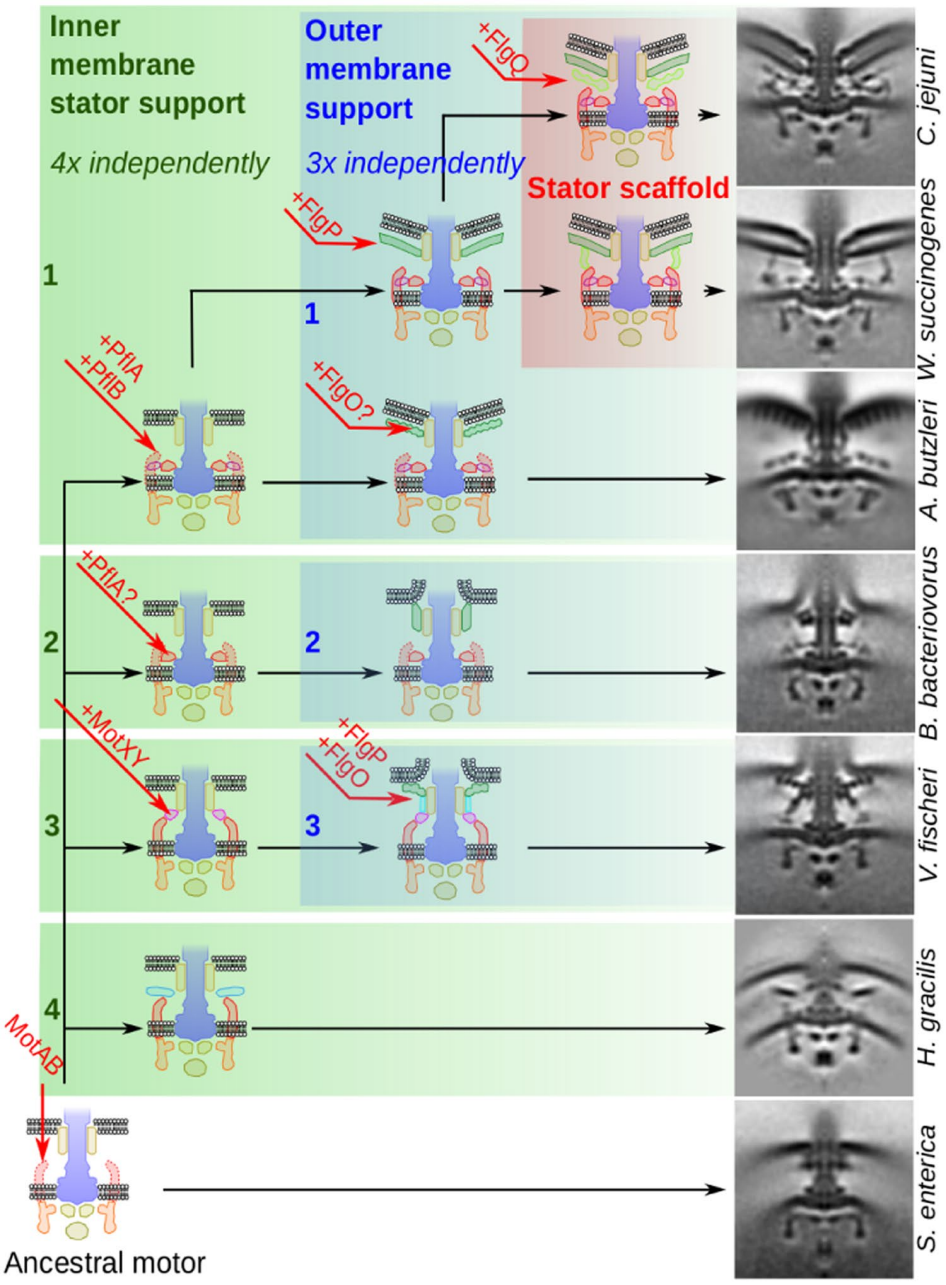
Proposed model of bacterial flagellar motor disk complex evolution. Evolving from a basic motor (bottom left), the incremental addition of proteins has on multiple independent occasions led to the assembly of inner membrane support structures (boxed in green) and outer membrane support structures (boxed in blue). In the ε-proteobacteria, these two structures have fused to form a scaffold structure (boxed in pink). Sub-tomogram averages on the right show contemporary motors that exemplify descendants of each ancestral intermediate.

The presence of PflA and PflB in the early-branching ε-proteobacterium *A. butzleri* motor suggests that the first step in evolution of the high-torque ε-proteobacterial motor was the incorporation of an initial periplasmic ring surrounding the rod co-localized with the stator complexes (Fig. 4). Indeed, analogous inner membrane-proximal stator support rings have evolved at least three times independently: the γ-proteobacterium *V. fischeri* features the MotXY-composed T-ring that engages with a ring of 13 stator complexes at high occupancy, the β-proteobacterium *Hylemonella gracilis* has convergently evolved an analogous ring composed of unknown proteins that supports a static ring of 13 likely stator complexes^2^, and the ε-proteobacteria feature PflA and PflB-based rings. In the more complex *Campylobacter-*type motors, these structures are required for stator complex binding, and deletion inactivates motor function^8^. Furthermore, the δ-proteobacteria species *B. bacteriovorus* has an inner membrane-proximal stator support ring that co-localizes with a ring of 12 likely stator complexes present at high occupancy. The protein(s) making this ring may be unrelated to the other three ring examples; alternatively it may be composed of a distant relative of the ε-proteobacterial PflA. We are now working to test this speculation. In addition to allowing us to propose that the first step in ε-proteobacterial motor evolution was incorporation of an inner membrane-associated ring, these observations also suggest that analogous inner membrane-proximal stator support rings have evolved at least three times independently, and thus are relatively ‘easy’ to evolve.

We propose that the wider rings composed of both PflA and PflB provide the selective benefit of higher torque by scaffolding wider rings of additional stator complexes. Indeed, these rings correlate with a “quantum leap” in the number of stator complexes incorporated into a motor. In motors without the PflB ring, the number of stators incorporated into the motor is 12 ± 1 (e.g., 11 in *S. enterica*, 12 in *B. bacteriovorus*, 13 in *V. fischeri* and *H. gracilis*), as compared to the 17 ± 1 stator complexes in ε-proteobacteria with the PflB proximal ring (e.g., 16 in *A. butzleri*, 17 in *C. jejuni* and *W. succinogenes*, and 18 in *H. pylori*). This increase in stator capacity is accompanied by an increase in torque-determining lever contact point distance of ~6 nm per stator. The increased torque-generation provided by this adaptation is significant and could potentially have contributed to members of the ε-proteobacteria moving into ecological niches of higher viscosity such as gut mucous. Indeed, *W. succinogenes* and *A. butzleri* are found in the animal gastrointestinal tract^12–14^ as is *C. jejuni*; *B. bacteriovorus* as a putative descendant of an intermediary state is sometimes found in the human gastrointestinal tract^15^. Addition of PflB and ultimately development of a full *Campylobacter-*type motor could have enabled descendants of this intermediary state to have made the gastrointestinal tract their sole niche.

We propose that the second step in evolution of the *Campylobacter-*type motor was the development of FlgP-based outer membrane basal disks as seen in *C. jejuni*, *W. succinogenes*, and the two *Helicobacter* species (Fig. 4). We propose, however, that the outer membrane basal disks were at first independent of the inner membrane-associated stator support rings. This speculation comes from the observation that FlgP can occur independent of PflA or PflB as demonstrated by the *Vibrio* motor. Indeed, as with inner membrane-associated rings, outer membrane-associated disks have evolved multiple times independently, and their presence does not always correspond to presence of PflA/B, or any of the analogous inner membrane-associated rings. For example, *B. bacteriovorus* features a small outer membrane-associated disk of unknown composition (Fig. 2), *V. fischeri* and *A. butzleri* feature larger disks of concentric rings, composed of FlgP and FlgO in *V*. *fischeri*, and suspected to contain at least FlgO, but not FlgP, in *A. butzleri*. Meanwhile, the *Campylobacter-*type motors feature large FlgP-based Archimedean spiral disks as seen in *C. jejuni*, *W. succinogenes*, and the *Helicobacters* (Fig. 2). The incongruency of the phylogeny of the *A. butzleri* motor and chimeric protein additions further highlights the modularity and independence of inner- and outer-membrane associated structures. The chimeric additions FlgH and FlgI form the P- and L-rings that serve as a platform for assembly of the outer membrane-disks, and their insertion into the *A. butzleri* motor, together with the accompanying non-FlgP disk, demonstrates the modularity of these components.

These outer membrane disks may have evolved as stabilizers for sheaths, or faster or higher-torque motors. As with inner membrane-associated stator support rings, their diversity highlights that these structures have evolved multiple times by incorporation of different proteins.

We propose the third step in evolution of the *Campylobacter-*type motor was fusion of the previously independent inner- and outer-membrane associated structures (Fig. 4). Inner- and outer-membrane associated rings and disks have evolved multiple times and can function independently. Specific to components of the ε-proteobacterial motors, FlgP-based outer membrane disks and PflA/B based inner membrane-associated stator support rings can occur independent of each other. For example, the *Vibrio* system features a FlgO/P based outer membrane disk but not PflA/B; conversely *Arcobacter* features PflA/B-based inner membrane-associated rings but not the FlgP-based outer membrane disk. This selective benefit of this fusion of the two independent structures systems may have been to stabilize high-occupancy binding of stator complexes irrespective of load on the motor, as all *Campylobacter-*type motors (*C. jejuni*, *W. succinogenes*, and *H. pylori*, with the exception of the *H. hepaticus* motor structure which is of insufficient resolution) have high occupancy stator complexes (Fig. 2), which may contribute to higher torque across all conditions. Because all these species inhabit animal guts, high nutrient availability would mitigate any selective negative ramifications of continual high torque generation.

What were the events in evolution of a wider C-ring? This remains unclear, although the *A. butzleri* C-ring is an intriguing intermediate between the *S. enterica* C-ring and the wider ε-proteobacterial C-ring. We previously noted that the correspondence of stator and rotor diameters and stoichiometries is loose, enabling staggered, asynchronous evolution of the stator and rotor rings while retaining a functional motor^8^. The *A. butzleri* C-ring appears to be a descendant of just such an intermediate, with an enlarged FliG ring yet retaining a narrower FliM/N ring. The *Campylobacter-*type C-ring incorporates additional proteins that we are yet to identify.

In addition to swim assay performance being consistent with motor structures, evolution of higher-torque motors is echoed by bacterial cell shape. Species with high torque motors have cell shapes more suited to motility in non-Newtonian viscous environments. *A. butzleri*, with its “intermediate” motor, has slightly curved, crescent-shaped cells, while *Campylobacter-*type motor organisms *C. jejuni*, *W. succinogenes*, *and Helicobacter* species have more prominently helical cell shapes. The increased swimming speeds of wild type *C. jejuni* relative to its straight mutant counterpart in methylcellulose demonstrate the clear benefit of a helical shape in this environment. This observation highlights the fact that evolution affects many aspects of cell biology and adaptation to higher viscosity environments can incorporate beneficial changes in cell size, shape and flagellar motor components simultaneously. We are now working to develop bead assays for different species to deconvolute effects of cell shape on motor output.

Why have not all bacteria evolved or acquired a high-torque motor such as the *Campylobacter-*type motor? This is likely a straightforward cost-benefit analysis, and organisms inhabiting nutrient rich, high viscosity environments such as many ε-proteobacteria will clearly benefit from such a motor. Other organisms that dwell in lower viscosity, lower nutrient environments might be more competitive to retain a simpler motor that is more efficient in its environment. Indeed, the ability to dynamically incorporate stator complexes as a function of load may be selectively beneficial unless the bacterium is continually exposed to high nutrient availability. Why *S. enterica*, which along with *C. jejuni* is a gut pathogen does not have a *Campylobacter-*type motor may be a historical accident, and *S. enterica* has never had an opportunity to receive a *Campylobacter-*type motor by horizontal gene transfer, or may reflect the selective benefit of dynamic stator complexes to *S. enterica*’s more varied lifestyle. Furthermore it is difficult to assess the contribution of motor torque and configuration for optimal chemotaxis.

In conclusion, our structural and phenotypic results enable us to propose a working model for the incremental evolution of the bacterial flagellar motor. Specifically, our results indicate that evolution of inner- and outer-membrane associated structures is both facile and independent, and in the case of the ε-proteobacteria these two systems fused to form the contemporary *Campylobacter-*type motor. Our results establish a structural basis to commence more focused studies to determine the identities of the novel accessory proteins that are incorporated into intermediary motors and trace where these accessories proteins originated from. Ultimately, these investigations will provide a more in-depth understanding of the evolution of molecular machines.

### Note added in proof

After this paper was accepted for publication, a recent paper was published that may form the basis for understanding different stator complex motor binding in different organisms with the observation that *E. coli* stator complexes binding behaves as a catch bond: Nord et al., PNAS 114(49):12952-12957 (2017). Relative stator complex on- and off- rates may be modulated in higher-torque motors to increase stator complex occupancy.

## Materials and Methods

### Protein sequences

All the protein sequences used in this analysis were retrieved from GenBank (National Center for Biotechnology Information (NCBI)), with accession numbers of each listed in Supplementary Tables S1 (core and accessory flagellar motor proteins) and **2** (ribosomal proteins). Ribosomal protein sequences (L1, L2, L3, L4, L5, L9, L10, L11, L13, L14, L15, L16, L17, L18) and core flagellar motor proteins (FlgB, FlgC, FlhB, FliE, FliF, FliG, FliI, FliM, FliN, FliP, FliR) were identified by BLASTP search from *Campylobacter jejuni* or *Escherichia coli* sequences. Accessory motor protein homologs (FlgO, FlgP, FlgQ, PflA, PflB) were identified by BLASTP search from *Campylobacter jejuni* (FlgQ, FlgP, PflA), *Vibrio chloerae* (FlgO, FlgP), and *Acetonema longum* (FlgP) sequences.

### Phylogenetic analysis

Core flagellar motor proteins and ribosomal proteins were aligned using Fast Statistical Alignment (FSA) (v1.1.5.9)^20^. Phylogenetically informative positions were determined from the alignments and extracted using the transitive consistency score (TCS) implementation within t-coffee (v11.00.d27cadf)^21^ and concatenated trees had individual protein alignments concatenated using seaview^22^. Phylogenetic inference was made using maximum-likelihood in GARLI (v2.01.1067)^23^, using Jones, Taylor and Thornton (JTT) amino acid substitution rates^24^. Trees were generated as the best of ten replicates with a termination criteria of 100,000 generations without a topology improvement of 0.0001 to the lnL score. Bootstrap sampling was done 1000 times with a termination criteria of 10,000 generations without a topology improvement of 0.01 in the lnL score for the concatenated trees and 100 times with a termination criterial of 1,000 generations without a topology improvement of 0.01 in the lnL score for the individual protein trees. Bootstrap support was converted to percentage and added to the base trees using SumTrees^25^ and visualized with FigTree (http://tree.bio.ed.ac.uk, v1.4.2, 2006–2014, Tree Figure Drawing Tool, Andrew Rambaut).

### Bacterial growth

*Campylobacter jejuni* 81–176 and *Arcobacter butzleri* DSM 7301 were grown from freezer stocks for 48 h on Mueller-Hinton agar (Fluka Analytical) or Columbia agar with 5% sheep’s blood (E&O Laboratories Ltd) under microaerophilic conditions using CampyPak sachets (Oxoid) at 37 °C. Cultures were subcultured, incubated for a further 16 h and collected from plates into ∼1.5 mL tryptone soya broth (TSB) (Oxoid). *Wolinella succinogenes* DSM 1740 was grown from freezer stocks anaerobically overnight in Hungate tubes containing DSMZ Medium 157 (DSMZ). *Salmonella enterica* serovar Typhimurium 12023 and *Bdellovibrio bacteriovorus* HID13 were grown from freezer stocks aerobically overnight in Luria-Bertani (LB) broth at 37 °C or Peptone-Yeast extract broth (10 g peptone, 3 g yeast extract per L; PY) at 30 °C, respectively, subcultured into media for 4 h additional growth and collected by centrifugation. All cultures were adjusted to an OD600 of ∼15.0 for electron cryo-tomography or OD600 of ~1.0 for transmission electron microscopy and light microscopy.

### Cryo-electron tomography and subtomogram averaging

Sample preparation, data collection and reconstruction were similar to^8^ with minor modifications. Briefly, Quantifoil R2/2 (200-mesh), UltrAu Foil Au200 R2/2 (Quantifoil Micro Tools GmbH) or C-flat CF-4/2-2C-50 (Electron Microscopy Sciences) grids were glow-discharged (60 s at 10 mA). Approximately 8 × 10^10^ 10-nm colloidal gold particles (Sigma) were added per 2.5 uL of cell suspension, with this volume being applied to the prepared grids and plunge frozen by a liquid ethane-propane mixture using a FEI Mark III or IV Vitrobot (FEI Company). Sample grids were then stored under liquid nitrogen until data collection.

Tomography tilt series were collected on a 200-kV FEI Tecnai G2 F20 TWIN (FEG) transmission electron microscope with a Direct Electron Detector (Falcon II) (FEI Company) and an overnight refill system^8^ using Gatan 914 or 626 cryo-holders. Tilt series were recorded from −54° to +54°, in 3° increments, between −3 μm and −8 μm defocus using Leginon automated data-collection software^26,27^. Images were collected at a nominal magnification of 25,000x to a final pixel size of 0.81 nm from a cumulative dose of ∼ 120e^−^/Å^2^.

Tomograms were reconstructed using RAPTOR^28^ and the IMOD package (IMOD v4.8.41, PEET v1.10.1, eTomo v4.8.41)^29^. Flagellar motors were manually picked and aligned from tomograms using PEET. FSC curves were determined from the final averages using PEET’s calcFSC (Supplementary Fig. S4). Isosurfaces were generated using UCSF Chimera.

### Negative stain electron microscopy

Bacterial cultures were deposited on glow discharged (30 s at 10 mA), 300 mesh, 3 mm copper grids with carbon support film (Taab Laboratory Equipment Ltd.) and negative stained with 2% uranyl acetate solution. Grids were imaged on a FEI Tecnai G2 Spirit BioTWIN (Tungsten) transmission electron microscope with a CCD camera (Eagle). Cell size was measured from collected images by determining the length (line drawn through middle of the cell, segmented for curved and helical cells) and width (across middle of cell) of 114–223 cells/species used in the viscosity experiments with ImageJ (v1.50 e)/Fiji software^30,31^. Differences in cell length and width measurements were evaluated by a one-way ANOVA, as implemented in SPSS (IBM Corp. Released 2013. IBM SPSS Statistics for Windows, Version 22.0. Armonk, NY: IBM Corp.).

### Light microscopy and swimming speed determination

To test bacterial swimming speeds in different viscosity solutions, Ficoll PM400 (Sigma) or methylcellulose 400 cP (Sigma) was dissolved in RF buffer (0.1 M potassium phosphate buffer (pH 7.0), 0.01 M sodium pyruvate, 2% Tween 80) to various concentrations. To calculate the dynamic viscosity of each solution (in cP) under experimental conditions, each solution was diluted one part media to three parts viscosity solution and the appropriately sized viscometers (Cannon-Fenske) and hydrometer (Amarell) were used to measure the kinematic viscosity of each solution (in cSt) and specific gravity, respectively. Dynamic viscosity (cP) was calculated as kinematic viscosity (cSt) × specific gravity.

For light microscopy cell tracking, bacterial cultures were mixed one part suspended bacterial culture to three parts viscosity solution (to give an cell OD600 of ~0.25), placed on a glass slide with double-sided tape on the edges to create a chamber, topped with a cover slip and viewed at 100x oil immersion on a MT4000 series light microscope (Meiji Techno) with a DMK23G618 digital camera connected to IC Capture software (v2.3.394.1917) (The Imaging Source). Bacterial cells were tracked using particle tracking software^32^ with ImageJ (v1.50 e)/Fiji software^30,31^ and converted to swimming velocities using an in-house script. Each point in the final graph represents the average of at least 600 bacterial cell swimming speeds, with >100 bacterial cells/video, 3 videos/viscosity, with the entire experiment done in duplicate. Error bars indicate ± one standard deviation.

### Data availability

The electron cryo-tomography subtomogram average density maps reported in this paper have been deposited in the Electron Microscopy Data Bank (EMD) (accession nos. *Wolinella succinogenes*: EMD-3912, *Arcobacter butzleri:* EMD-3910, *Bdellovibrio bacteriovorus:* EMD-3911).

## Electronic supplementary material

Supplemental Table 1

Supplemental Table 2

Supplemental Figures
