# Supplementary material for: Evolution of higher torque in Campylobacter-type bacterial flagellar motors
Source: Sci Rep. 2018 Jan 8;8:97. doi: 10.1038/s41598-017-18115-1 (PMC5758724; doi:10.1038/s41598-017-18115-1)
Supplement: Supplementary file 3 — Supplemental Figures [file 41598_2017_18115_MOESM3_ESM.pdf]

Supplemental Information

"Evolution of higher torque in *Campylobacter*-type bacterial flagellar motors"

Bonnie Chaban[1†], Izaak Coleman[1], Morgan Beeby[1]*

[1]Department of Life Sciences, Imperial College of London, London, SW7 2AZ, UK

*corresponding author: MB: mbeeby@imperial.ac.uk, phone +44 (0) 20 7594 5251

[†]Current address: Faculty of Science, Health, Education and Engineering, University of the Sunshine Coast, 90 Sippy Downs Drive, Sippy Downs, 4556, QLD, Australia

Supplementary Figures S1, S2, S3 and S4 (attached)

Supplemental Information

"Evolution of higher torque in *Campylobacter*-type bacterial flagellar motors"

Bonnie Chaban[1†], Izaak Coleman[1], Morgan Beeby[1]*

[1]Department of Life Sciences, Imperial College of London, London, SW7 2AZ, UK

*corresponding author: MB: mbeeby@imperial.ac.uk, phone +44 (0) 20 7594 5251

[†]Current address: Faculty of Science, Health, Education and Engineering, University of the Sunshine Coast, 90 Sippy Downs Drive, Sippy Downs, 4556, QLD, Australia

Supplementary Figure S1: Flagellar motor phylogeny with a focus on ε-proteobacteria and related species. Phylogeny was determined using a concatenation of core flagellar motor proteins FlgB, FlgC, FlhB, FliE, FliF, FliG, FliI, FliM, FliN, FliP, FliR, bootstrapped 1000 times.

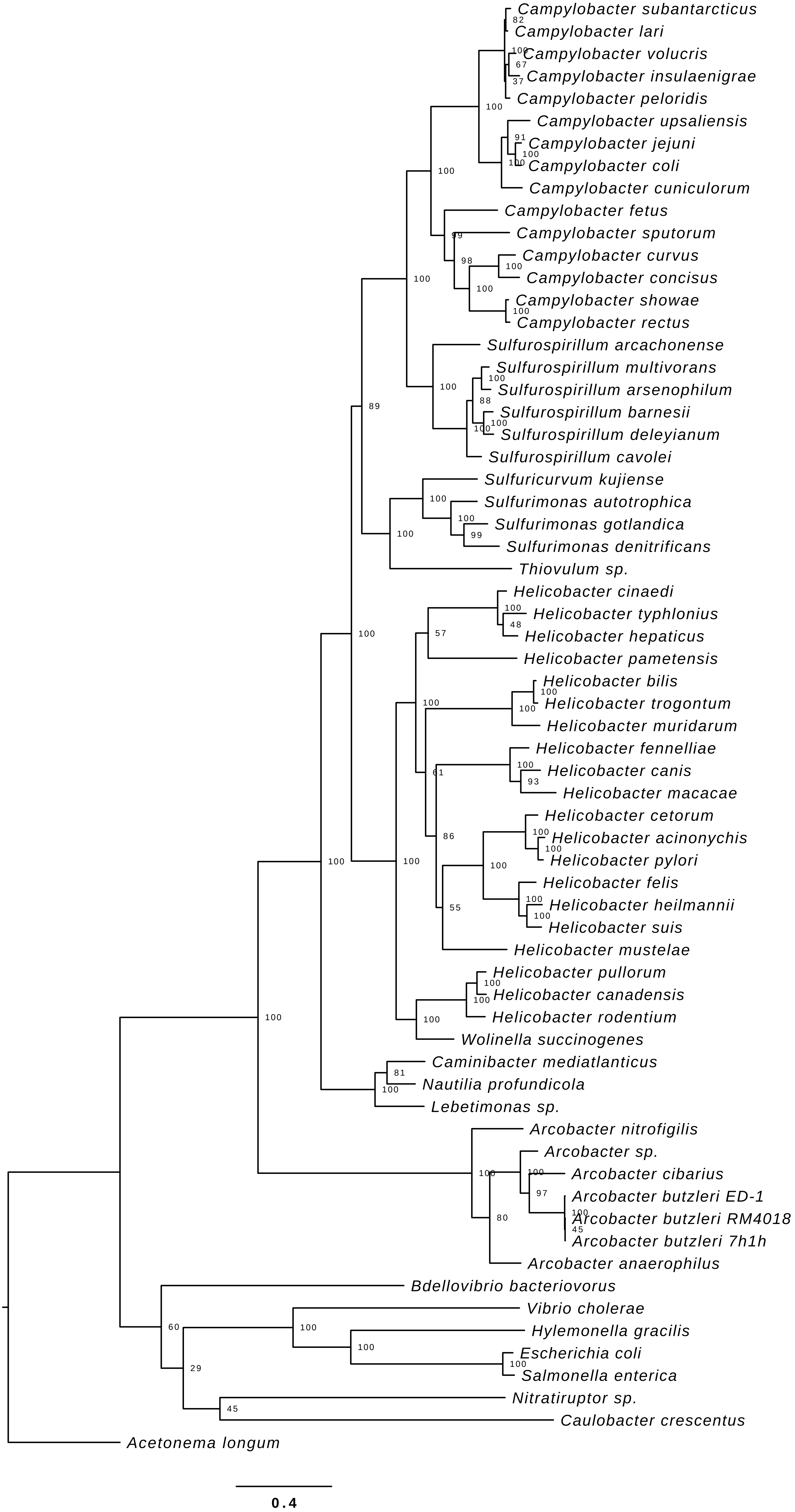
Campylobacter subantarcticus
Campylobacter lari
Campylobacter volucris
Campylobacter insulaenigrae
Campylobacter peloridis
Campylobacter upsaliensis
Campylobacter jejuni
Campylobacter coli
Campylobacter cuniculorum
Campylobacter fetus
Campylobacter sputorum
Campylobacter curvus
Campylobacter concisus
Campylobacter showae
Campylobacter rectus
Sulfurospirillum arcachonense
Sulfurospirillum multivorans
Sulfurospirillum arsenophilum
Sulfurospirillum barnesii
Sulfurospirillum deleyianum
Sulfurospirillum cavolei
Sulfuricurvum kujiense
Sulfurimonas autotrophica
Sulfurimonas gotlandica
Sulfurimonas denitrificans
Thiovulum sp.
Helicobacter cinaedi
Helicobacter typhlonius
Helicobacter hepaticus
Helicobacter pametensis
Helicobacter bilis
Helicobacter trogontum
Helicobacter muridarum
Helicobacter fennelliae
Helicobacter canis
Helicobacter macacae
Helicobacter cetorum
Helicobacter acinonychis
Helicobacter pylori
Helicobacter felis
Helicobacter heilmannii
Helicobacter suis
Helicobacter mustelae
Helicobacter pullorum
Helicobacter canadensis
Helicobacter rodentium
Wolinella succinogenes
Caminibacter mediatlanticus
Nautilia profundicola
Lebetimonas sp.
Arcobacter nitrofigilis
Arcobacter sp.
Arcobacter cibarius
Arcobacter butzleri ED-1
Arcobacter butzleri RM4018
Arcobacter butzleri 7h1h
Arcobacter anaerophilus
Bdellovibrio bacteriovorus
Vibrio cholerae
Hylemonella gracilis
Escherichia coli
Salmonella enterica
Nitratiruptor sp.
Caulobacter crescentus
Acetonema longum
0.4

Supplemental Information

"Evolution of higher torque in *Campylobacter*-type bacterial flagellar motors"

Bonnie Chaban[1†], Izaak Coleman[1], Morgan Beeby[1]*

[1]Department of Life Sciences, Imperial College of London, London, SW7 2AZ, UK

*corresponding author: MB: mbeeby@imperial.ac.uk, phone +44 (0) 20 7594 5251

[†]Current address: Faculty of Science, Health, Education and Engineering, University of the Sunshine Coast, 90 Sippy Downs Drive, Sippy Downs, 4556, QLD, Australia

Supplementary Figure S2: Organism phylogeny of organisms depicted in Figure S1. Phylogeny was determined using a concatenation of ribosomal protein sequences L1, L2, L3, L4, L5, L9, L10, L11, L13, L14, L15, L16, L17, L18, bootstrapped 1000 times.

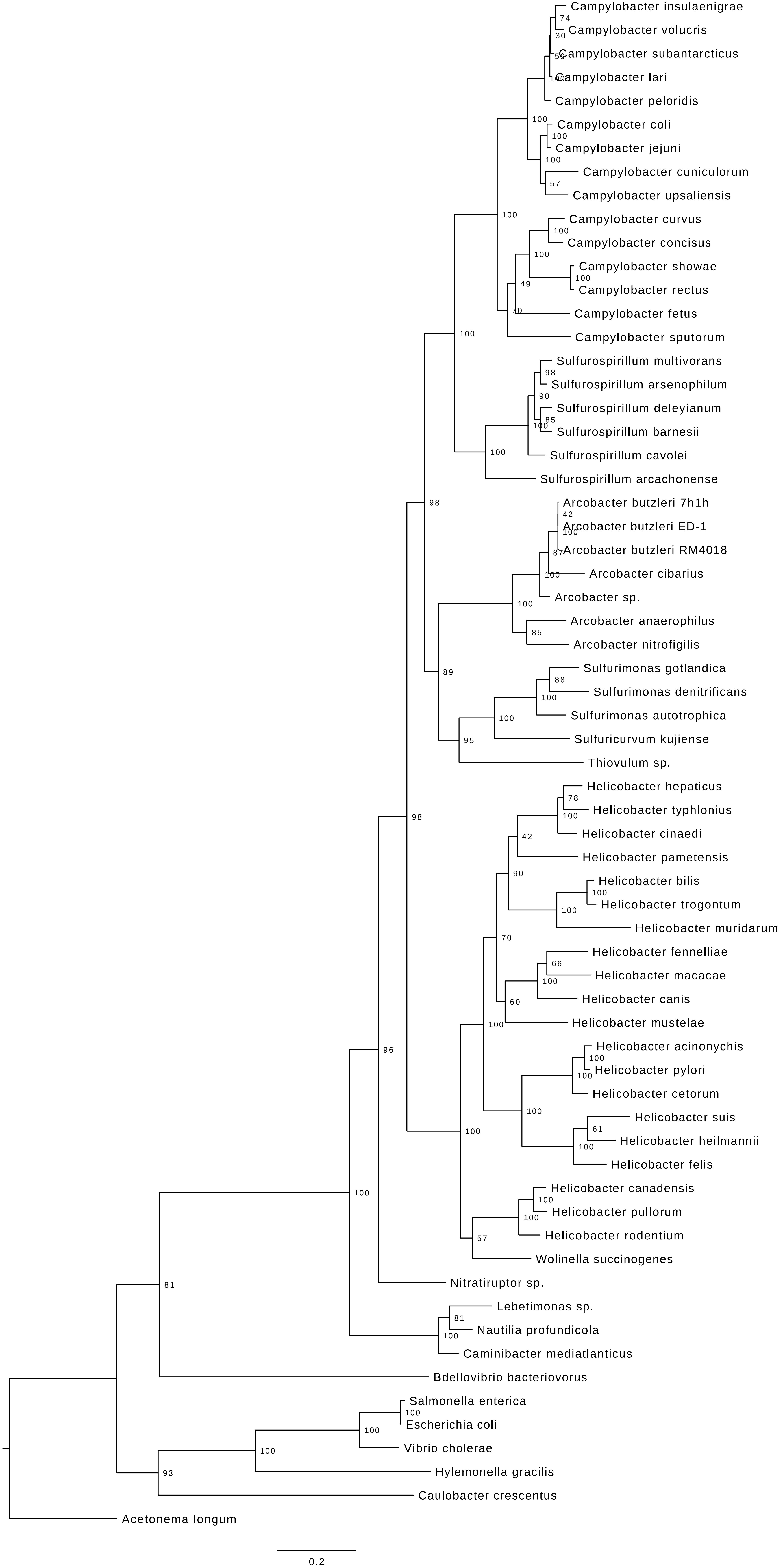

Campylobacter insulaenigrae
Campylobacter volucris
Campylobacter subantarcticus
Campylobacter lari
Campylobacter peloridis
Campylobacter coli
Campylobacter jejuni
Campylobacter cuniculorum
Campylobacter upsaliensis
Campylobacter curvus
Campylobacter concisus
Campylobacter showae
Campylobacter rectus
Campylobacter fetus
Campylobacter sputorum
Sulfurospirillum multivorans
Sulfurospirillum arsenophilum
Sulfurospirillum deleyianum
Sulfurospirillum barnesii
Sulfurospirillum cavolei
Sulfurospirillum arcachonense
Arcobacter butzleri 7h1h
Arcobacter butzleri ED-1
Arcobacter butzleri RM4018
Arcobacter cibarius
Arcobacter sp.
Arcobacter anaerophilus
Arcobacter nitrofigilis
Sulfurimonas gotlandica
Sulfurimonas denitrificans
Sulfurimonas autotrophica
Sulfuricurvum kujiense
Thiovulum sp.
Helicobacter hepaticus
Helicobacter typhlonius
Helicobacter cinaedi
Helicobacter pametensis
Helicobacter bilis
Helicobacter trogontum
Helicobacter muridarum
Helicobacter fennelliae
Helicobacter macacae
Helicobacter canis
Helicobacter mustelae
Helicobacter acinonychis
Helicobacter pylori
Helicobacter cetorum
Helicobacter suis
Helicobacter heilmannii
Helicobacter felis
Helicobacter canadensis
Helicobacter pullorum
Helicobacter rodentium
Wolinella succinogenes
Nitratiruptor sp.
Lebetimonas sp.
Nautilia profundicola
Caminibacter mediatlanticus
Bdellovibrio bacteriovorus
Salmonella enterica
Escherichia coli
Vibrio cholerae
Hylemonella gracilis
Caulobacter crescentus
Acetonema longum
0.2

Supplemental Information

"Evolution of higher torque in *Campylobacter*-type bacterial flagellar motors"

Bonnie Chaban[1†], Izaak Coleman[1], Morgan Beeby[1]*

[1]Department of Life Sciences, Imperial College of London, London, SW7 2AZ, UK

*corresponding author: MB: mbeeby@imperial.ac.uk, phone +44 (0) 20 7594 5251

[†]Current address: Faculty of Science, Health, Education and Engineering, University of the Sunshine Coast, 90 Sippy Downs Drive, Sippy Downs, 4556, QLD, Australia

Supplementary Figure S3: Flagellar motor phylogeny of each flagellar motor protein separately, with a focus on ε-proteobacteria and related species. Phylogeny was determined for FlgB, FlgC, FlgH, FlgI, FlhA, FlhB, FliE, FliF, FliG, FliI, FliM, FliN, FliP, FliQ, FliR, individually, bootstrapped 100 times.

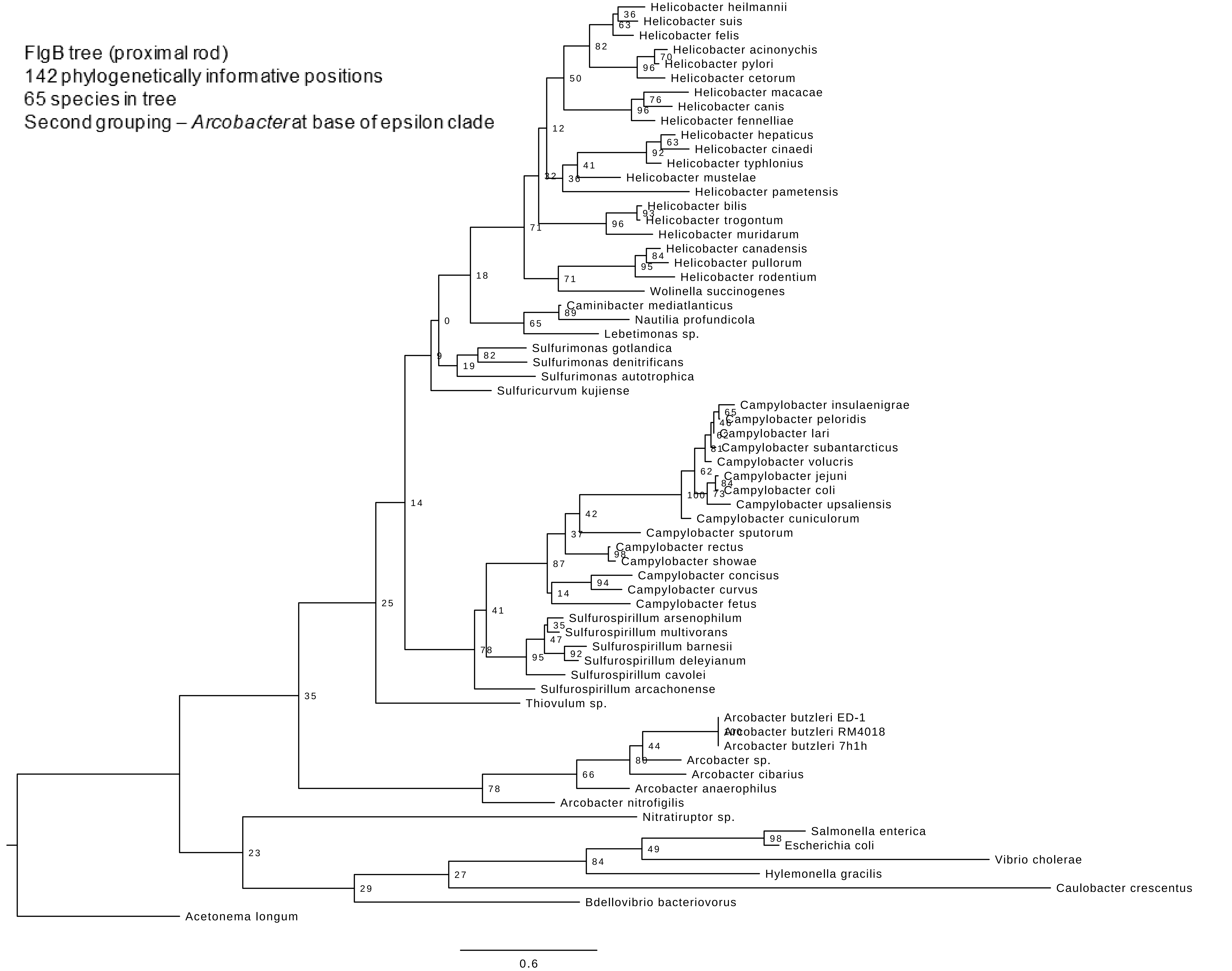
FlgB tree (proximal rod)
142 phylogenetically informative positions
65 species in tree
Second grouping – *Arcobacter* at base of epsilon clade
Helicobacter heilmannii
Helicobacter suis
Helicobacter felis
Helicobacter acinonychis
Helicobacter pylori
Helicobacter cetorum
Helicobacter macacae
Helicobacter canis
Helicobacter fennelliae
Helicobacter hepaticus
Helicobacter cinaedi
Helicobacter typhlonius
Helicobacter mustelae
Helicobacter pametensis
Helicobacter bilis
Helicobacter trogontum
Helicobacter muridarum
Helicobacter canadensis
Helicobacter pullorum
Helicobacter rodentium
Wolinella succinogenes
Caminibacter mediatlanticus
Nautilia profundicola
Lebetimonas sp.
Sulfurimonas gotlandica
Sulfurimonas denitrificans
Sulfurimonas autotrophica
Sulfuricurvum kujiense
Campylobacter insulaenigrae
Campylobacter peloridis
Campylobacter lari
Campylobacter subantarcticus
Campylobacter volucris
Campylobacter jejuni
Campylobacter coli
Campylobacter upsaliensis
Campylobacter cuniculorum
Campylobacter sputorum
Campylobacter rectus
Campylobacter showae
Campylobacter concisus
Campylobacter curvus
Campylobacter fetus
Sulfurospirillum arsenophilum
Sulfurospirillum multivorans
Sulfurospirillum barnesii
Sulfurospirillum deleyianum
Sulfurospirillum cavolei
Sulfurospirillum arcachonense
Thiovulum sp.
Arcobacter butzleri ED-1
Arcobacter butzleri RM4018
Arcobacter butzleri 7h1h
Arcobacter sp.
Arcobacter cibarius
Arcobacter anaerophilus
Arcobacter nitrofigilis
Nitratiruptor sp.
Salmonella enterica
Escherichia coli
Vibrio cholerae
Hylemonella gracilis
Caulobacter crescentus
Bdellovibrio bacteriovorus
Acetonema longum
0.6

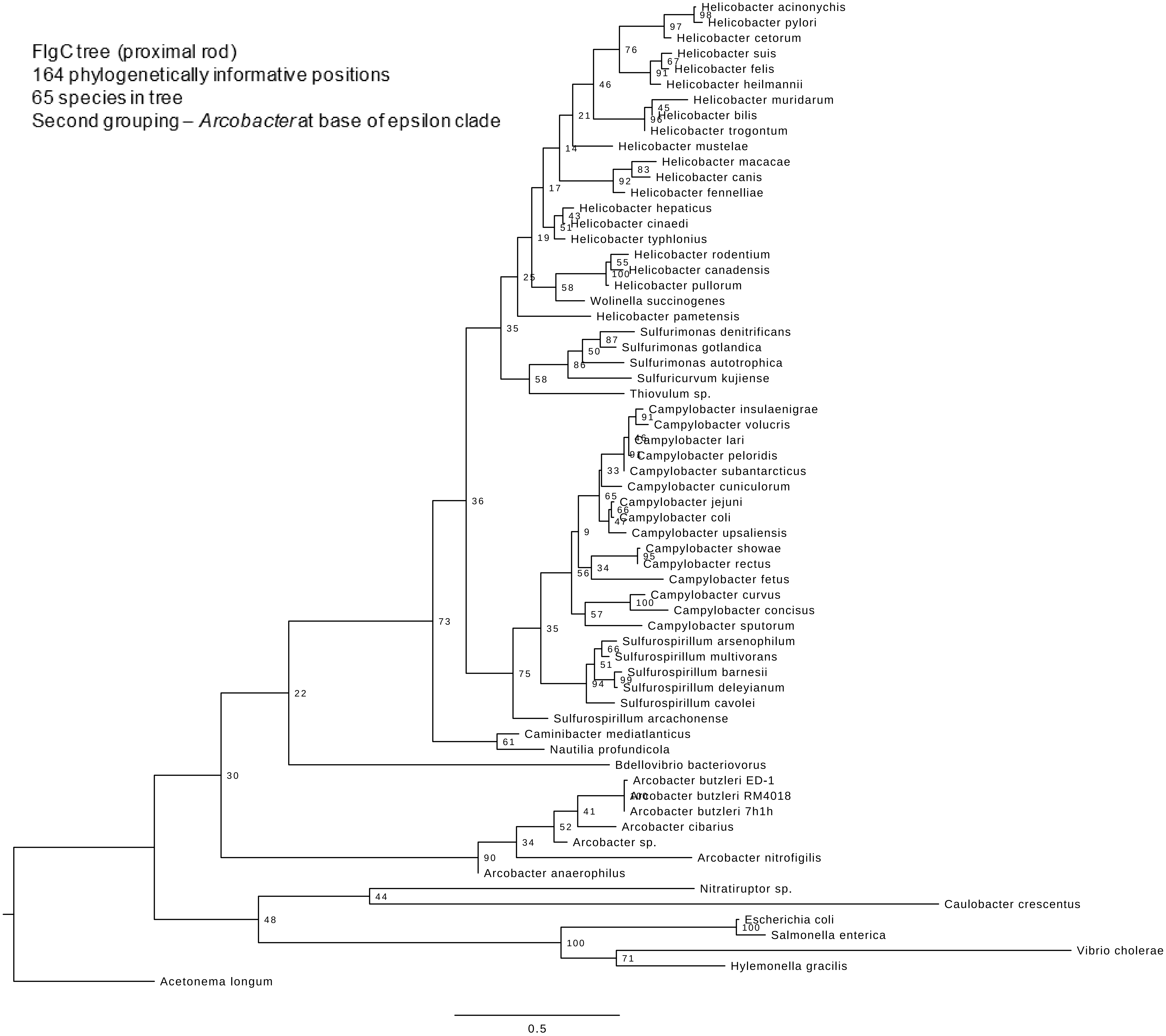
FlgC tree (proximal rod)
164 phylogenetically informative positions
65 species in tree
Second grouping – *Arcobacter* at base of epsilon clade
Helicobacter acinonychis
Helicobacter pylori
Helicobacter cetorum
Helicobacter suis
Helicobacter felis
Helicobacter heilmannii
Helicobacter muridarum
Helicobacter bilis
Helicobacter trogontum
Helicobacter mustelae
Helicobacter macacae
Helicobacter canis
Helicobacter fennelliae
Helicobacter hepaticus
Helicobacter cinaedi
Helicobacter typhlonius
Helicobacter rodentium
Helicobacter canadensis
Helicobacter pullorum
Wolinella succinogenes
Helicobacter pametensis
Sulfurimonas denitrificans
Sulfurimonas gotlandica
Sulfurimonas autotrophica
Sulfuricurvum kujiense
Thiovulum sp.
Campylobacter insulaenigrae
Campylobacter volucris
Campylobacter lari
Campylobacter peloridis
Campylobacter subantarcticus
Campylobacter cuniculorum
Campylobacter jejuni
Campylobacter coli
Campylobacter upsaliensis
Campylobacter showae
Campylobacter rectus
Campylobacter fetus
Campylobacter curvus
Campylobacter concisus
Campylobacter sputorum
Sulfurospirillum arsenophilum
Sulfurospirillum multivorans
Sulfurospirillum barnesii
Sulfurospirillum deleyianum
Sulfurospirillum cavolei
Sulfurospirillum arcachonense
Caminibacter mediatlanticus
Nautilia profundicola
Bdellovibrio bacteriovorus
Arcobacter butzleri ED-1
Arcobacter butzleri RM4018
Arcobacter butzleri 7h1h
Arcobacter cibarius
Arcobacter sp.
Arcobacter nitrofigilis
Arcobacter anaerophilus
Nitratiruptor sp.
Caulobacter crescentus
Escherichia coli
Salmonella enterica
Vibrio cholerae
Hylemonella gracilis
Acetonema longum
0.5

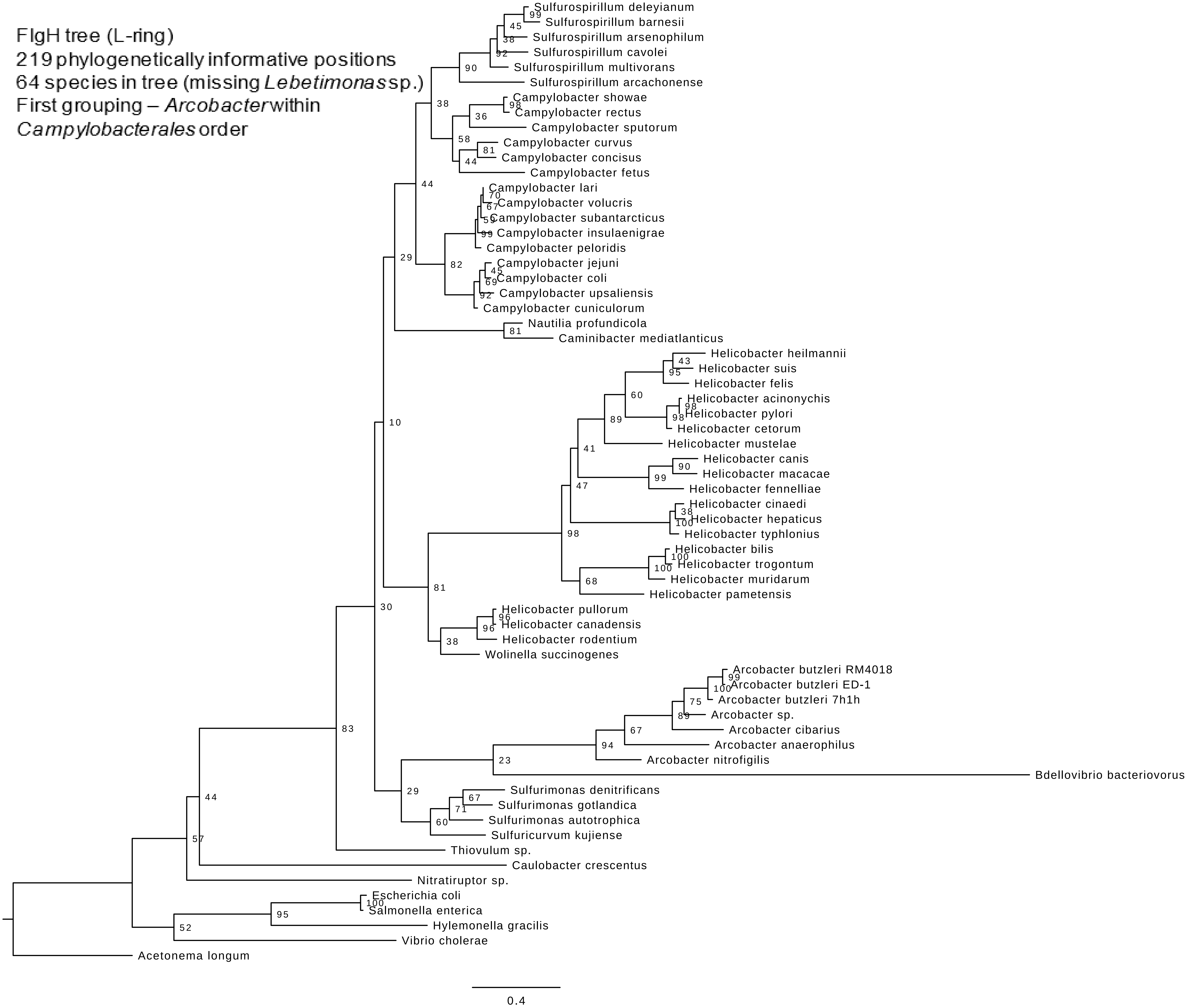
FlgH tree (L-ring)
219 phylogenetically informative positions
64 species in tree (missing *Lebetimonas* sp.)
First grouping – *Arcobacter* within
Campylobacterales order
Sulfurospirillum deleyianum
Sulfurospirillum barnesii
Sulfurospirillum arsenophilum
Sulfurospirillum cavolei
Sulfurospirillum multivorans
Sulfurospirillum arcachonense
Campylobacter showae
Campylobacter rectus
Campylobacter sputorum
Campylobacter curvus
Campylobacter concisus
Campylobacter fetus
Campylobacter lari
Campylobacter volucris
Campylobacter subantarcticus
Campylobacter insulaenigrae
Campylobacter peloridis
Campylobacter jejuni
Campylobacter coli
Campylobacter upsaliensis
Campylobacter cuniculorum
Nautilia profundicola
Caminibacter mediatlanticus
Helicobacter heilmannii
Helicobacter suis
Helicobacter felis
Helicobacter acinonychis
Helicobacter pylori
Helicobacter cetorum
Helicobacter mustelae
Helicobacter canis
Helicobacter macacae
Helicobacter fennelliae
Helicobacter cinaedi
Helicobacter hepaticus
Helicobacter typhlonius
Helicobacter bilis
Helicobacter trogontum
Helicobacter muridarum
Helicobacter pametensis
Helicobacter pullorum
Helicobacter canadensis
Helicobacter rodentium
Wolinella succinogenes
Arcobacter butzleri RM4018
Arcobacter butzleri ED-1
Arcobacter butzleri 7h1h
Arcobacter sp.
Arcobacter cibarius
Arcobacter anaerophilus
Arcobacter nitrofigilis
Bdellovibrio bacteriovorus
Sulfurimonas denitrificans
Sulfurimonas gotlandica
Sulfurimonas autotrophica
Sulfuricurvum kujiense
Thiovulum sp.
Caulobacter crescentus
Nitratiruptor sp.
Escherichia coli
Salmonella enterica
Hylemonella gracilis
Vibrio cholerae
Acetonema longum
0.4

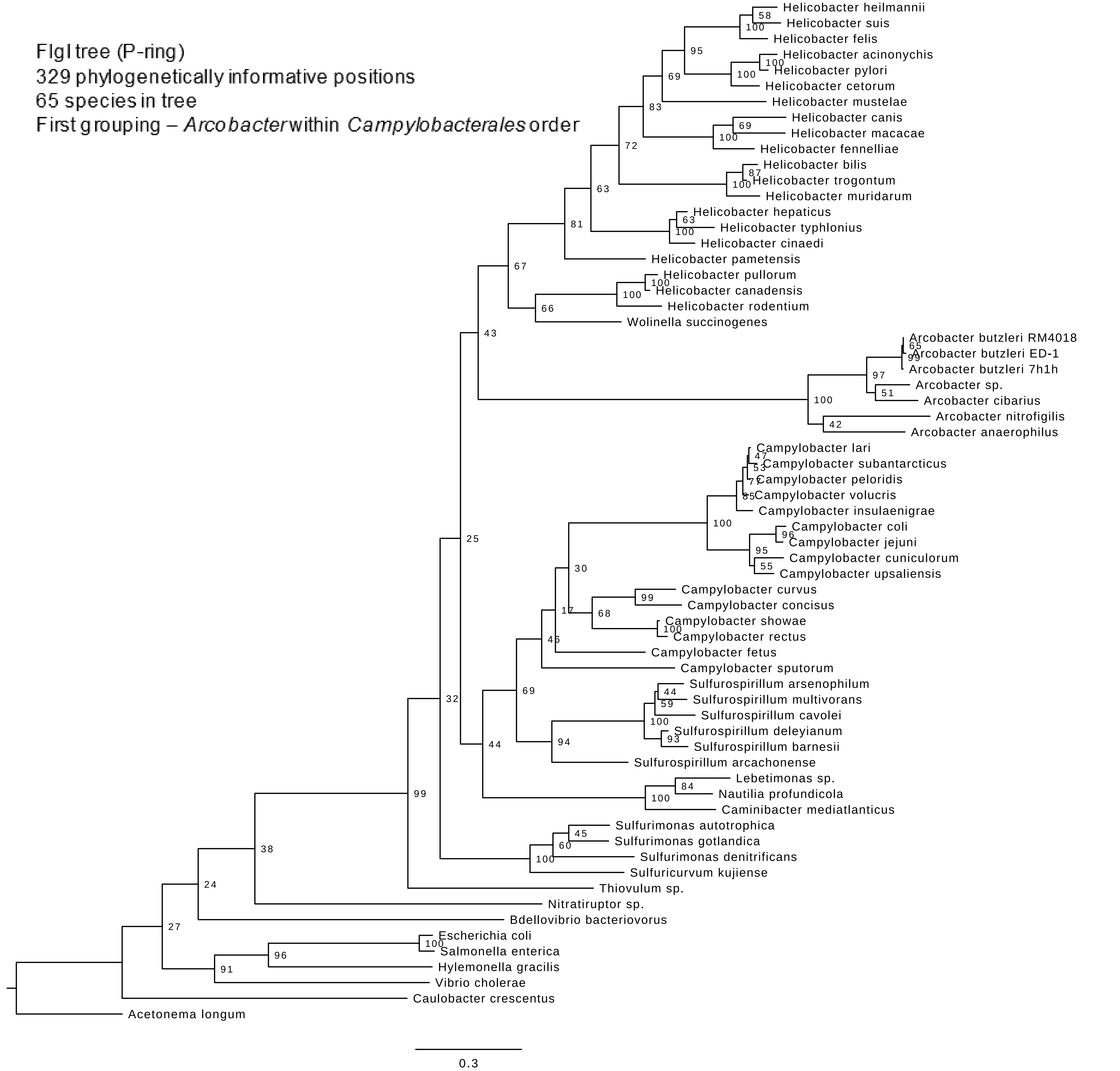
FlgI tree (P-ring)
329 phylogenetically informative positions
65 species in tree
First grouping – *Arcobacter* within *Campylobacterales* order
Helicobacter heilmannii
Helicobacter suis
Helicobacter felis
Helicobacter acinonychis
Helicobacter pylori
Helicobacter cetorum
Helicobacter mustelae
Helicobacter canis
Helicobacter macacae
Helicobacter fennelliae
Helicobacter bilis
Helicobacter trogontum
Helicobacter muridarum
Helicobacter hepaticus
Helicobacter typhlonius
Helicobacter cinaedi
Helicobacter pametensis
Helicobacter pullorum
Helicobacter canadensis
Helicobacter rodentium
Wolinella succinogenes
Arcobacter butzleri RM4018
Arcobacter butzleri ED-1
Arcobacter butzleri 7h1h
Arcobacter sp.
Arcobacter cibarius
Arcobacter nitrofigilis
Arcobacter anaerophilus
Campylobacter lari
Campylobacter subantarcticus
Campylobacter peloridis
Campylobacter volucris
Campylobacter insulaenigrae
Campylobacter coli
Campylobacter jejuni
Campylobacter cuniculorum
Campylobacter upsaliensis
Campylobacter curvus
Campylobacter concisus
Campylobacter showae
Campylobacter rectus
Campylobacter fetus
Campylobacter sputorum
Sulfurospirillum arsenophilum
Sulfurospirillum multivorans
Sulfurospirillum cavolei
Sulfurospirillum deleyianum
Sulfurospirillum barnesii
Sulfurospirillum arcachonense
Lebetimonas sp.
Nautilia profundicola
Caminibacter mediatlanticus
Sulfurimonas autotrophica
Sulfurimonas gotlandica
Sulfurimonas denitrificans
Sulfuricurvum kujiense
Thiovulum sp.
Nitratiruptor sp.
Bdellovibrio bacteriovorus
Escherichia coli
Salmonella enterica
Hylemonella gracilis
Vibrio cholerae
Caulobacter crescentus
Acetonema longum
0.3

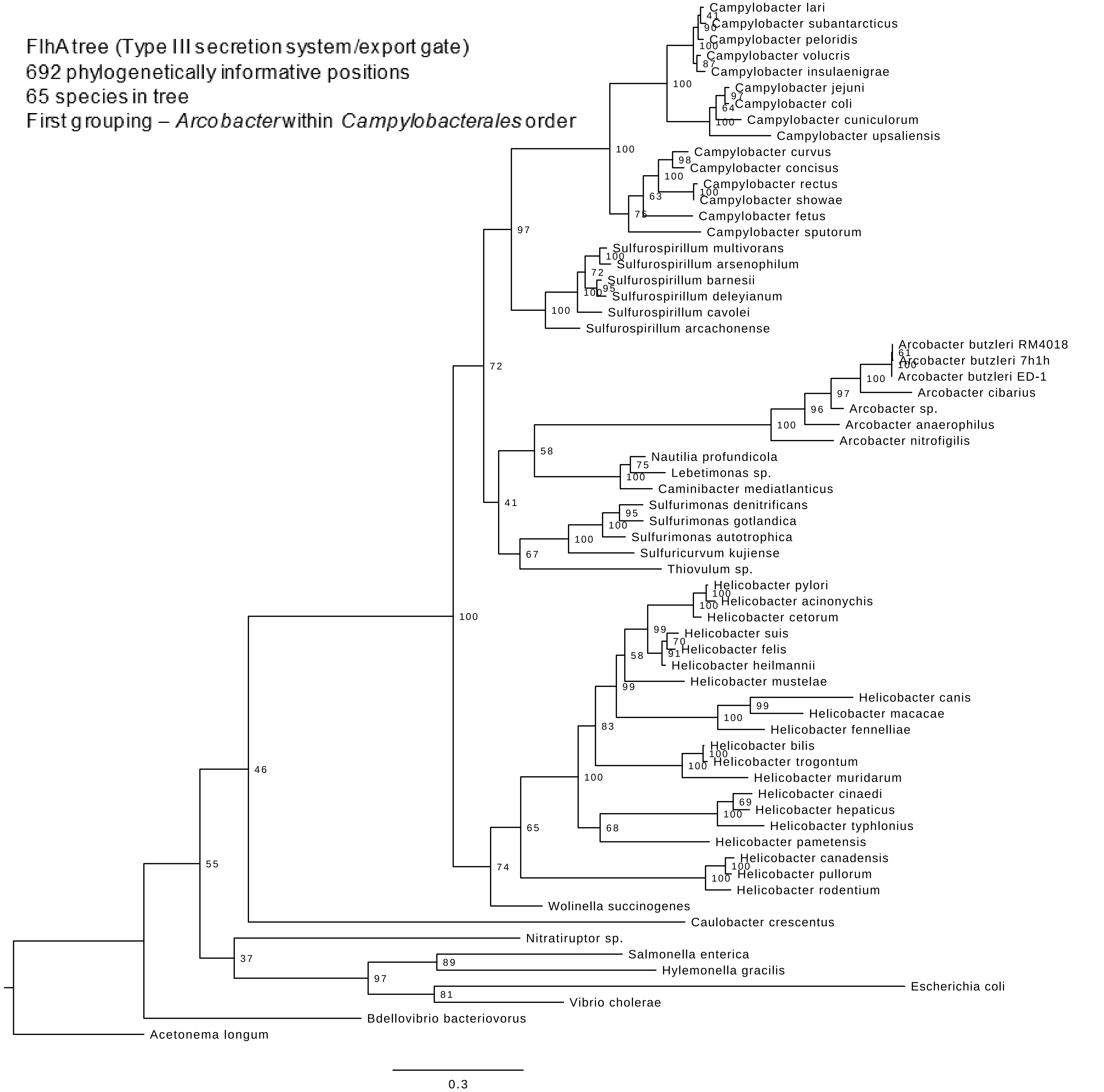

FlhA tree (Type III secretion system/export gate)
692 phylogenetically informative positions
65 species in tree
First grouping – Arcobacter within Campylobacterales order
Campylobacter lari
Campylobacter subantarcticus
Campylobacter peloridis
Campylobacter volucris
Campylobacter insulaenigrae
Campylobacter jejuni
Campylobacter coli
Campylobacter cuniculorum
Campylobacter upsaliensis
Campylobacter curvus
Campylobacter concisus
Campylobacter rectus
Campylobacter showae
Campylobacter fetus
Campylobacter sputorum
Sulfurospirillum multivorans
Sulfurospirillum arsenophilum
Sulfurospirillum barnesii
Sulfurospirillum deleyianum
Sulfurospirillum cavolei
Sulfurospirillum arcachonense
Arcobacter butzleri RM4018
Arcobacter butzleri 7h1h
Arcobacter butzleri ED-1
Arcobacter cibarius
Arcobacter sp.
Arcobacter anaerophilus
Arcobacter nitrofigilis
Nautilia profundicola
Lebetimonas sp.
Caminibacter mediatlanticus
Sulfurimonas denitrificans
Sulfurimonas gotlandica
Sulfurimonas autotrophica
Sulfuricurvum kujiense
Thiovulum sp.
Helicobacter pylori
Helicobacter acinonychis
Helicobacter cetorum
Helicobacter suis
Helicobacter felis
Helicobacter heilmannii
Helicobacter mustelae
Helicobacter canis
Helicobacter macacae
Helicobacter fennelliae
Helicobacter bilis
Helicobacter trogontum
Helicobacter muridarum
Helicobacter cinaedi
Helicobacter hepaticus
Helicobacter typhlonius
Helicobacter pametensis
Helicobacter canadensis
Helicobacter pullorum
Helicobacter rodentium
Wolinella succinogenes
Caulobacter crescentus
Nitratiruptor sp.
Salmonella enterica
Hylemonella gracilis
Escherichia coli
Vibrio cholerae
Bdellovibrio bacteriovorus
Acetonema longum
0.3

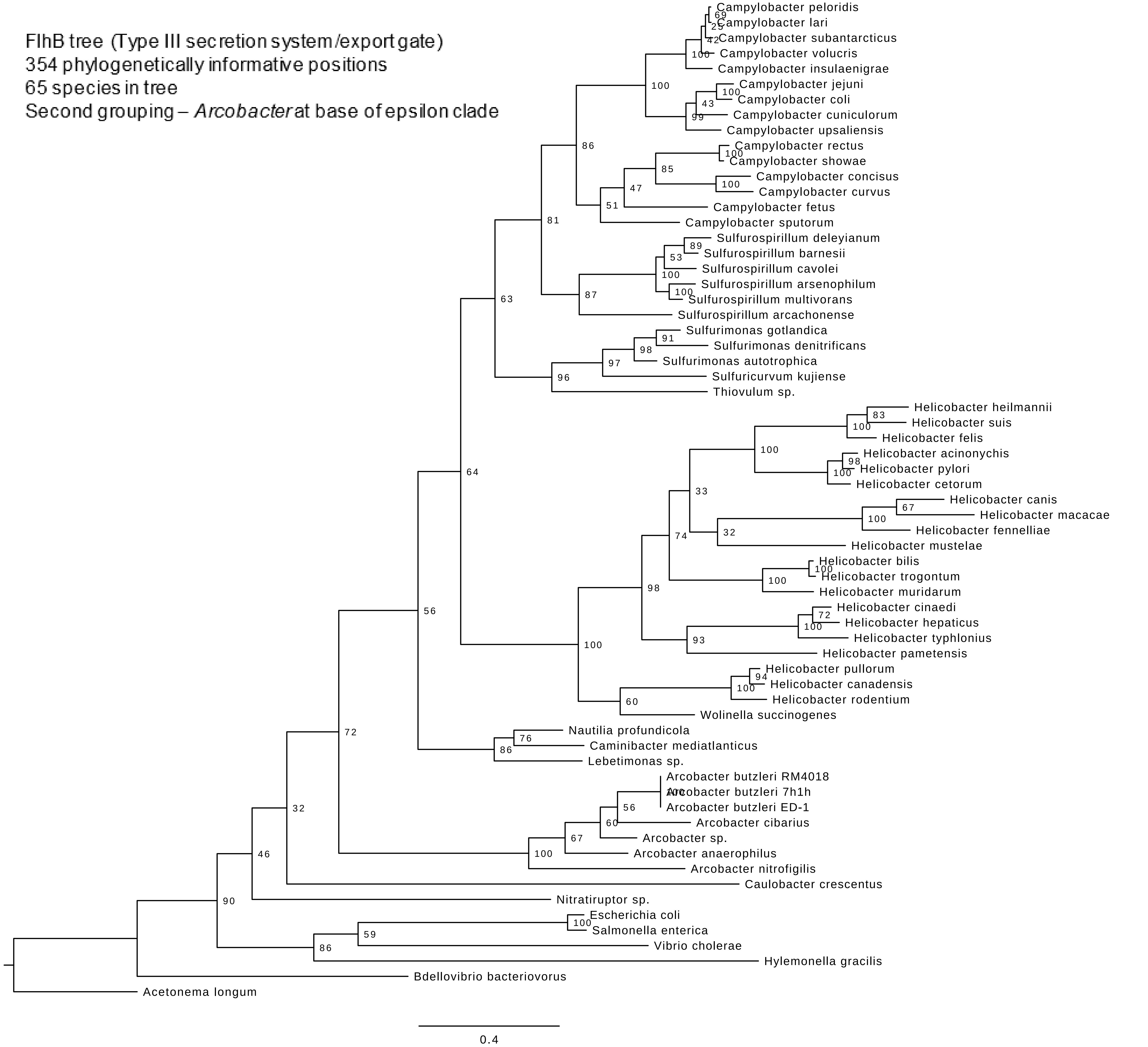
FlhB tree (Type III secretion system/export gate)
354 phylogenetically informative positions
65 species in tree
Second grouping – *Arcobacter* at base of epsilon clade
Campylobacter peloridis
Campylobacter lari
Campylobacter subantarcticus
Campylobacter volucris
Campylobacter insulaenigrae
Campylobacter jejuni
Campylobacter coli
Campylobacter cuniculorum
Campylobacter upsaliensis
Campylobacter rectus
Campylobacter showae
Campylobacter concisus
Campylobacter curvus
Campylobacter fetus
Campylobacter sputorum
Sulfurospirillum deleyianum
Sulfurospirillum barnesii
Sulfurospirillum cavolei
Sulfurospirillum arsenophilum
Sulfurospirillum multivorans
Sulfurospirillum arcachonense
Sulfurimonas gotlandica
Sulfurimonas denitrificans
Sulfurimonas autotrophica
Sulfuricurvum kujiense
Thiovulum sp.
Helicobacter heilmannii
Helicobacter suis
Helicobacter felis
Helicobacter acinonychis
Helicobacter pylori
Helicobacter cetorum
Helicobacter canis
Helicobacter macacae
Helicobacter fennelliae
Helicobacter mustelae
Helicobacter bilis
Helicobacter trogontum
Helicobacter muridarum
Helicobacter cinaedi
Helicobacter hepaticus
Helicobacter typhlonius
Helicobacter pametensis
Helicobacter pullorum
Helicobacter canadensis
Helicobacter rodentium
Wolinella succinogenes
Nautilia profundicola
Caminibacter mediatlanticus
Lebetimonas sp.
Arcobacter butzleri RM4018
Arcobacter butzleri 7h1h
Arcobacter butzleri ED-1
Arcobacter cibarius
Arcobacter sp.
Arcobacter anaerophilus
Arcobacter nitrofigilis
Caulobacter crescentus
Nitratiruptor sp.
Escherichia coli
Salmonella enterica
Vibrio cholerae
Hylemonella gracilis
Bdellovibrio bacteriovorus
Acetonema longum
0.4

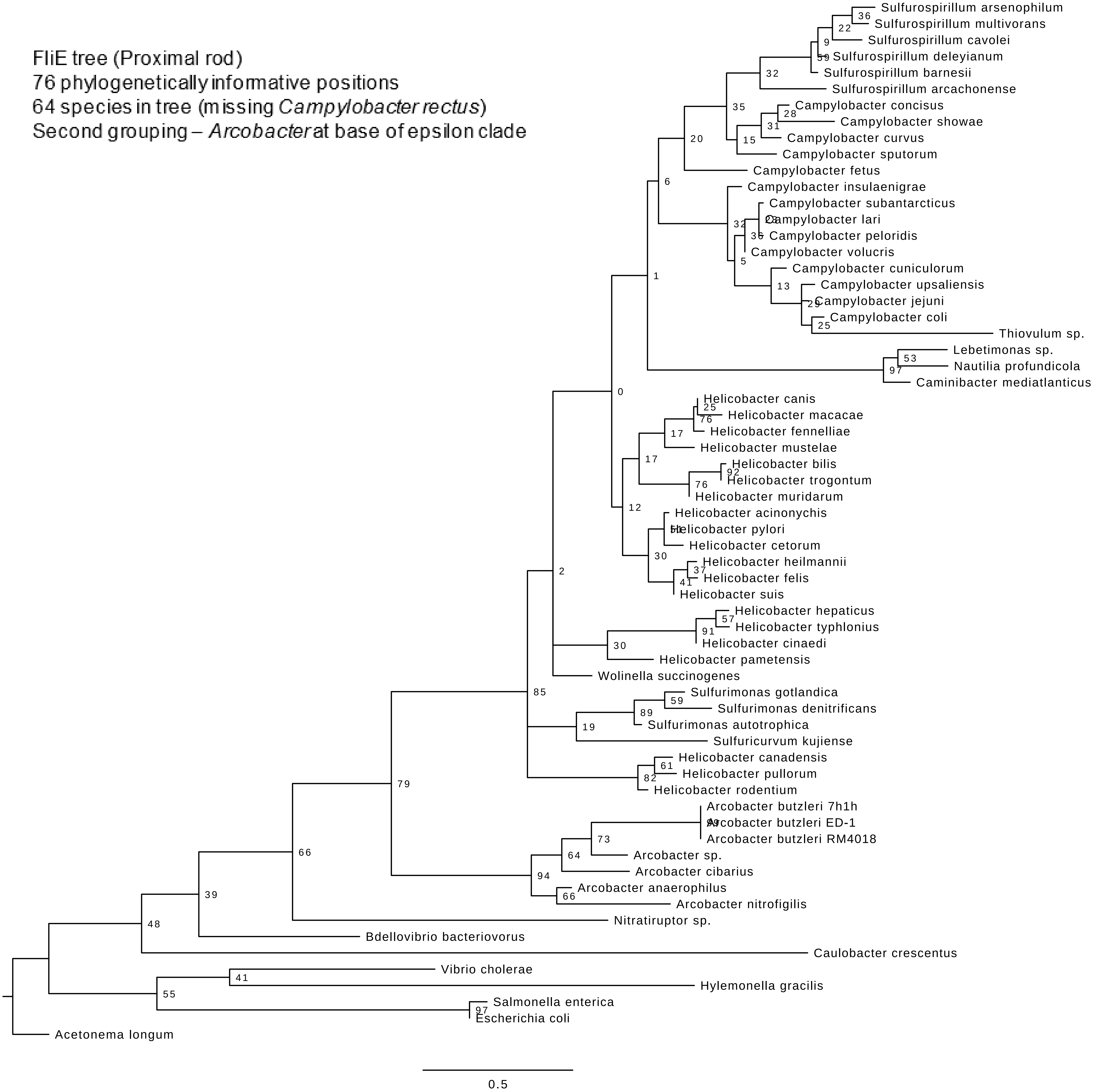
FliE tree (Proximal rod)
76 phylogenetically informative positions
64 species in tree (missing *Campylobacter rectus*)
Second grouping – *Arcobacter* at base of epsilon clade
Sulfurospirillum arsenophilum
Sulfurospirillum multivorans
Sulfurospirillum cavolei
Sulfurospirillum deleyianum
Sulfurospirillum barnesii
Sulfurospirillum arcachonense
Campylobacter concisus
Campylobacter showae
Campylobacter curvus
Campylobacter sputorum
Campylobacter fetus
Campylobacter insulaenigrae
Campylobacter subantarcticus
Campylobacter lari
Campylobacter peloridis
Campylobacter volucris
Campylobacter cuniculorum
Campylobacter upsaliensis
Campylobacter jejuni
Campylobacter coli
Thiovulum sp.
Lebetimonas sp.
Nautilia profundicola
Caminibacter mediatlanticus
Helicobacter canis
Helicobacter macacae
Helicobacter fennelliae
Helicobacter mustelae
Helicobacter bilis
Helicobacter trogontum
Helicobacter muridarum
Helicobacter acinonychis
Helicobacter pylori
Helicobacter cetorum
Helicobacter heilmannii
Helicobacter felis
Helicobacter suis
Helicobacter hepaticus
Helicobacter typhlonius
Helicobacter cinaedi
Helicobacter pametensis
Wolinella succinogenes
Sulfurimonas gotlandica
Sulfurimonas denitrificans
Sulfurimonas autotrophica
Sulfuricurvum kujiense
Helicobacter canadensis
Helicobacter pullorum
Helicobacter rodentium
Arcobacter butzleri 7h1h
Arcobacter butzleri ED-1
Arcobacter butzleri RM4018
Arcobacter sp.
Arcobacter cibarius
Arcobacter anaerophilus
Arcobacter nitrofigilis
Nitratiruptor sp.
Bdellovibrio bacteriovorus
Caulobacter crescentus
Vibrio cholerae
Hylemonella gracilis
Salmonella enterica
Escherichia coli
Acetonema longum

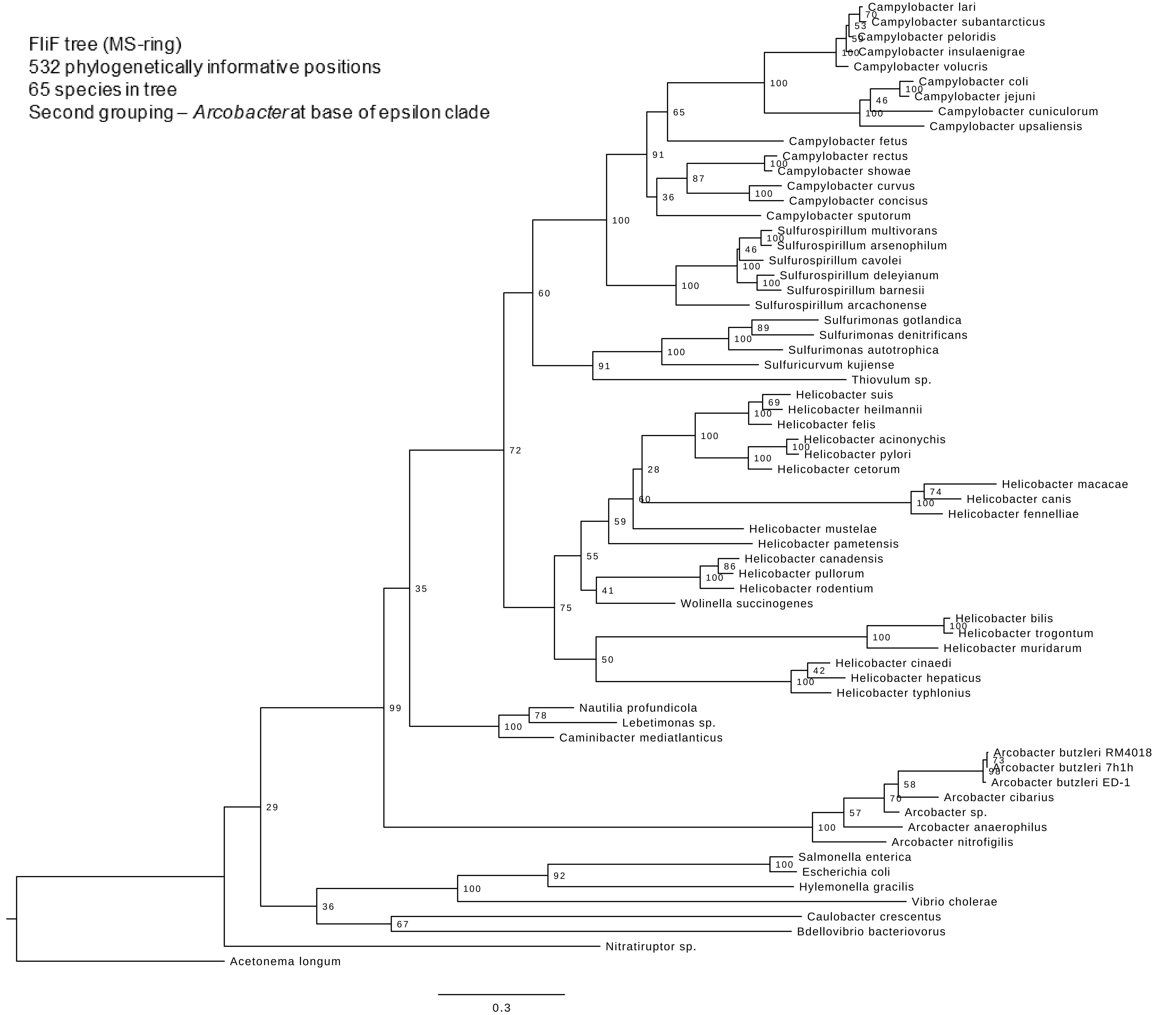
FliF tree (MS-ring)
532 phylogenetically informative positions
65 species in tree
Second grouping – Arcobacter at base of epsilon clade
Campylobacter lari
Campylobacter subantarcticus
Campylobacter peloridis
Campylobacter insulaenigrae
Campylobacter volucris
Campylobacter coli
Campylobacter jejuni
Campylobacter cuniculorum
Campylobacter upsaliensis
Campylobacter fetus
Campylobacter rectus
Campylobacter showae
Campylobacter curvus
Campylobacter concisus
Campylobacter sputorum
Sulfurospirillum multivorans
Sulfurospirillum arsenophilum
Sulfurospirillum cavolei
Sulfurospirillum deleyianum
Sulfurospirillum barnesii
Sulfurospirillum arcachonense
Sulfurimonas gotlandica
Sulfurimonas denitrificans
Sulfurimonas autotrophica
Sulfuricurvum kujiense
Thiovulum sp.
Helicobacter suis
Helicobacter heilmannii
Helicobacter felis
Helicobacter acinonychis
Helicobacter pylori
Helicobacter cetorum
Helicobacter macacae
Helicobacter canis
Helicobacter fennelliae
Helicobacter mustelae
Helicobacter pametensis
Helicobacter canadensis
Helicobacter pullorum
Helicobacter rodentium
Wolinella succinogenes
Helicobacter bilis
Helicobacter trogontum
Helicobacter muridarum
Helicobacter cinaedi
Helicobacter hepaticus
Helicobacter typhlonius
Nautilia profundicola
Lebetimonas sp.
Caminibacter mediatlanticus
Arcobacter butzleri RM4018
Arcobacter butzleri 7h1h
Arcobacter butzleri ED-1
Arcobacter cibarius
Arcobacter sp.
Arcobacter anaerophilus
Arcobacter nitrofigilis
Salmonella enterica
Escherichia coli
Hylemonella gracilis
Vibrio cholerae
Caulobacter crescentus
Bdellovibrio bacteriovorus
Nitratiruptor sp.
Acetonema longum
0.3

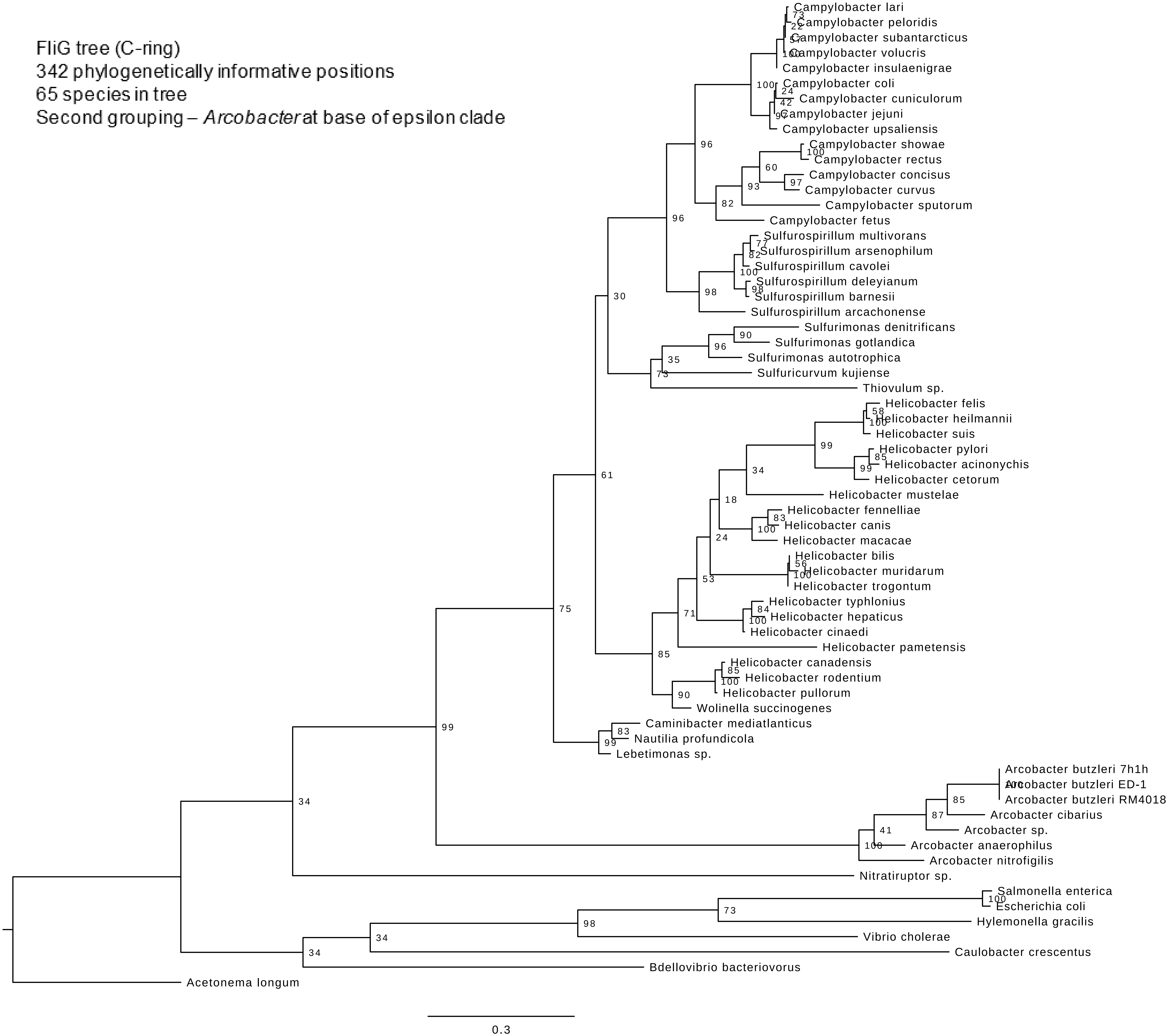
FliG tree (C-ring)
342 phylogenetically informative positions
65 species in tree
Second grouping – *Arcobacter* at base of epsilon clade
Campylobacter lari
Campylobacter peloridis
Campylobacter subantarcticus
Campylobacter volucris
Campylobacter insulaenigrae
Campylobacter coli
Campylobacter cuniculorum
Campylobacter jejuni
Campylobacter upsaliensis
Campylobacter showae
Campylobacter rectus
Campylobacter concisus
Campylobacter curvus
Campylobacter sputorum
Campylobacter fetus
Sulfurospirillum multivorans
Sulfurospirillum arsenophilum
Sulfurospirillum cavolei
Sulfurospirillum deleyianum
Sulfurospirillum barnesii
Sulfurospirillum arcachonense
Sulfurimonas denitrificans
Sulfurimonas gotlandica
Sulfurimonas autotrophica
Sulfuricurvum kujiense
Thiovulum sp.
Helicobacter felis
Helicobacter heilmannii
Helicobacter suis
Helicobacter pylori
Helicobacter acinonychis
Helicobacter cetorum
Helicobacter mustelae
Helicobacter fennelliae
Helicobacter canis
Helicobacter macacae
Helicobacter bilis
Helicobacter muridarum
Helicobacter trogontum
Helicobacter typhlonius
Helicobacter hepaticus
Helicobacter cinaedi
Helicobacter pametensis
Helicobacter canadensis
Helicobacter rodentium
Helicobacter pullorum
Wolinella succinogenes
Caminibacter mediatlanticus
Nautilia profundicola
Lebetimonas sp.
Arcobacter butzleri 7h1h
Arcobacter butzleri ED-1
Arcobacter butzleri RM4018
Arcobacter cibarius
Arcobacter sp.
Arcobacter anaerophilus
Arcobacter nitrofigilis
Nitratiruptor sp.
Salmonella enterica
Escherichia coli
Hylemonella gracilis
Vibrio cholerae
Caulobacter crescentus
Bdellovibrio bacteriovorus
Acetonema longum
0.3

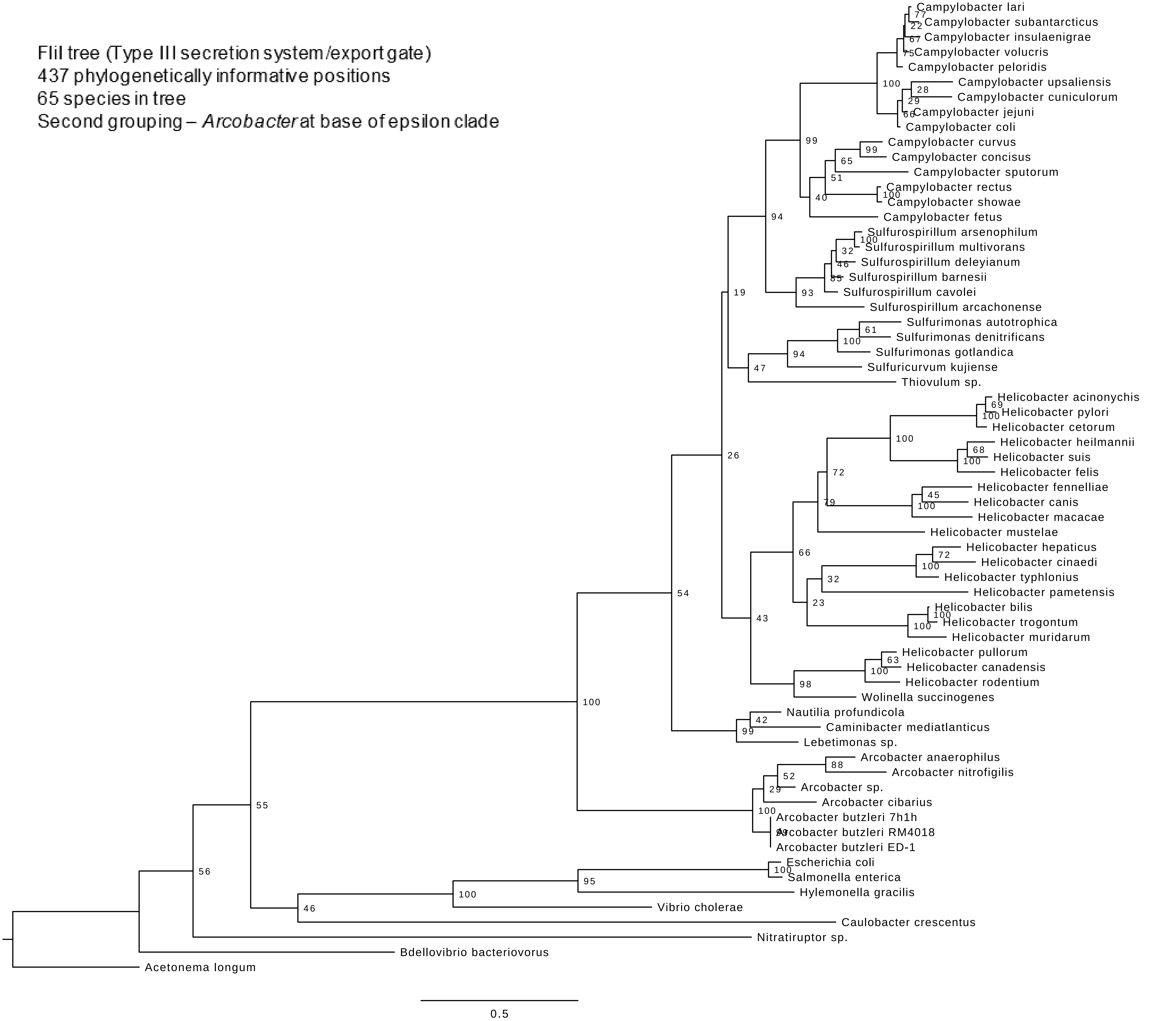
FliI tree (Type III secretion system/export gate)
437 phylogenetically informative positions
65 species in tree
Second grouping – *Arcobacter* at base of epsilon clade
Campylobacter lari
Campylobacter subantarcticus
Campylobacter insulaenigrae
Campylobacter volucris
Campylobacter peloridis
Campylobacter upsaliensis
Campylobacter cuniculorum
Campylobacter jejuni
Campylobacter coli
Campylobacter curvus
Campylobacter concisus
Campylobacter sputorum
Campylobacter rectus
Campylobacter showae
Campylobacter fetus
Sulfurospirillum arsenophilum
Sulfurospirillum multivorans
Sulfurospirillum deleyianum
Sulfurospirillum barnesii
Sulfurospirillum cavolei
Sulfurospirillum arcachonense
Sulfurimonas autotrophica
Sulfurimonas denitrificans
Sulfurimonas gotlandica
Sulfuricurvum kujiense
Thiovulum sp.
Helicobacter acinonychis
Helicobacter pylori
Helicobacter cetorum
Helicobacter heilmannii
Helicobacter suis
Helicobacter felis
Helicobacter fennelliae
Helicobacter canis
Helicobacter macacae
Helicobacter mustelae
Helicobacter hepaticus
Helicobacter cinaedi
Helicobacter typhlonius
Helicobacter pametensis
Helicobacter bilis
Helicobacter trogontum
Helicobacter muridarum
Helicobacter pullorum
Helicobacter canadensis
Helicobacter rodentium
Wolinella succinogenes
Nautilia profundicola
Caminibacter mediatlanticus
Lebetimonas sp.
Arcobacter anaerophilus
Arcobacter nitrofigilis
Arcobacter sp.
Arcobacter cibarius
Arcobacter butzleri 7h1h
Arcobacter butzleri RM4018
Arcobacter butzleri ED-1
Escherichia coli
Salmonella enterica
Hylemonella gracilis
Vibrio cholerae
Caulobacter crescentus
Nitratiruptor sp.
Bdellovibrio bacteriovorus
Acetonema longum
0.5

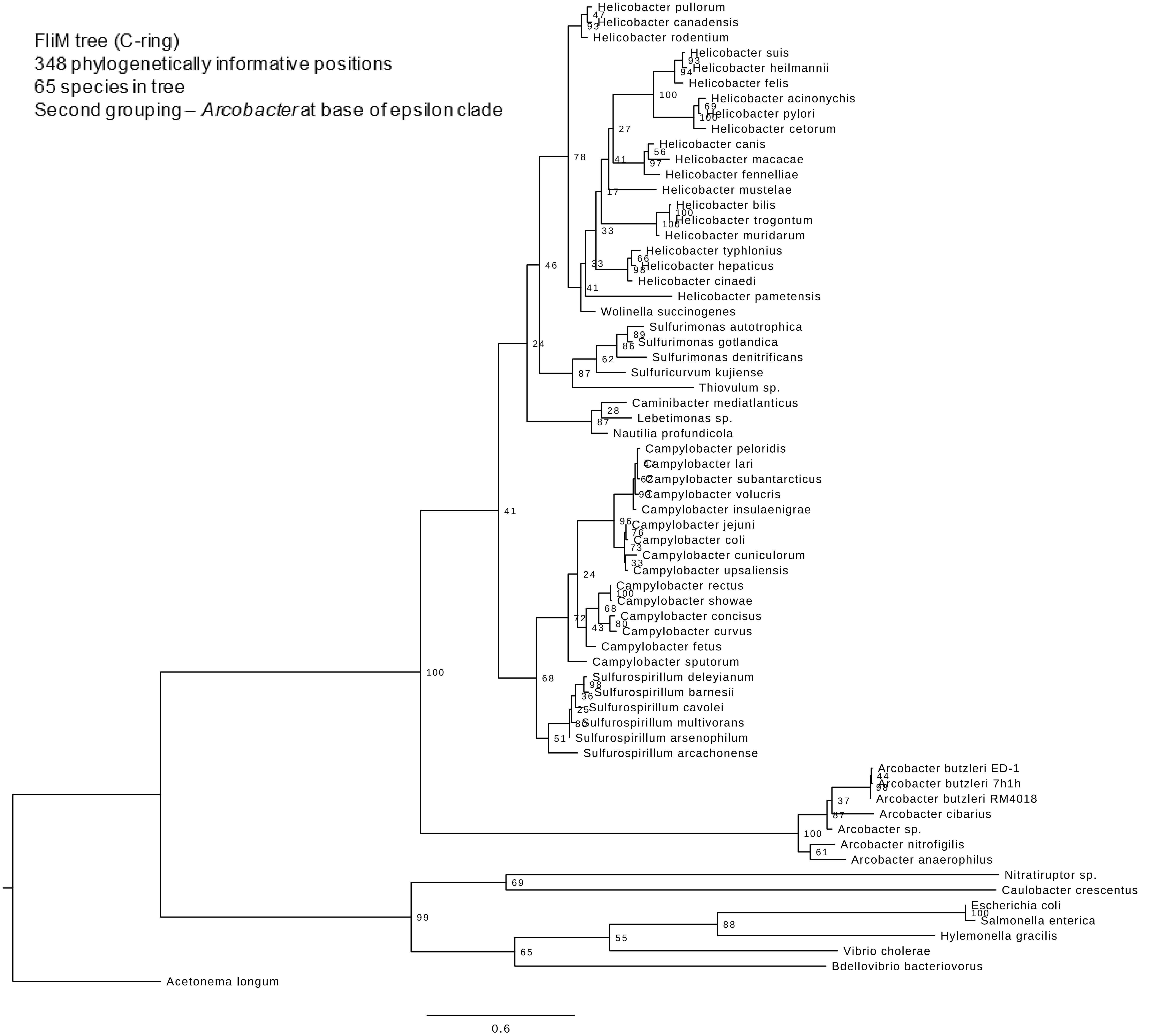
FliM tree (C-ring)
348 phylogenetically informative positions
65 species in tree
Second grouping – *Arcobacter* at base of epsilon clade
Helicobacter pullorum
Helicobacter canadensis
Helicobacter rodentium
Helicobacter suis
Helicobacter heilmannii
Helicobacter felis
Helicobacter acinonychis
Helicobacter pylori
Helicobacter cetorum
Helicobacter canis
Helicobacter macacae
Helicobacter fennelliae
Helicobacter mustelae
Helicobacter bilis
Helicobacter trogontum
Helicobacter muridarum
Helicobacter typhlonius
Helicobacter hepaticus
Helicobacter cinaedi
Helicobacter pametensis
Wolinella succinogenes
Sulfurimonas autotrophica
Sulfurimonas gotlandica
Sulfurimonas denitrificans
Sulfuricurvum kujiense
Thiovulum sp.
Caminibacter mediatlanticus
Lebetimonas sp.
Nautilia profundicola
Campylobacter peloridis
Campylobacter lari
Campylobacter subantarcticus
Campylobacter volucris
Campylobacter insulaenigrae
Campylobacter jejuni
Campylobacter coli
Campylobacter cuniculorum
Campylobacter upsaliensis
Campylobacter rectus
Campylobacter showae
Campylobacter concisus
Campylobacter curvus
Campylobacter fetus
Campylobacter sputorum
Sulfurospirillum deleyianum
Sulfurospirillum barnesii
Sulfurospirillum cavolei
Sulfurospirillum multivorans
Sulfurospirillum arsenophilum
Sulfurospirillum arcachonense
Arcobacter butzleri ED-1
Arcobacter butzleri 7h1h
Arcobacter butzleri RM4018
Arcobacter cibarius
Arcobacter sp.
Arcobacter nitrofigilis
Arcobacter anaerophilus
Nitratiruptor sp.
Caulobacter crescentus
Escherichia coli
Salmonella enterica
Hylemonella gracilis
Vibrio cholerae
Bdellovibrio bacteriovorus
Acetonema longum
0.6

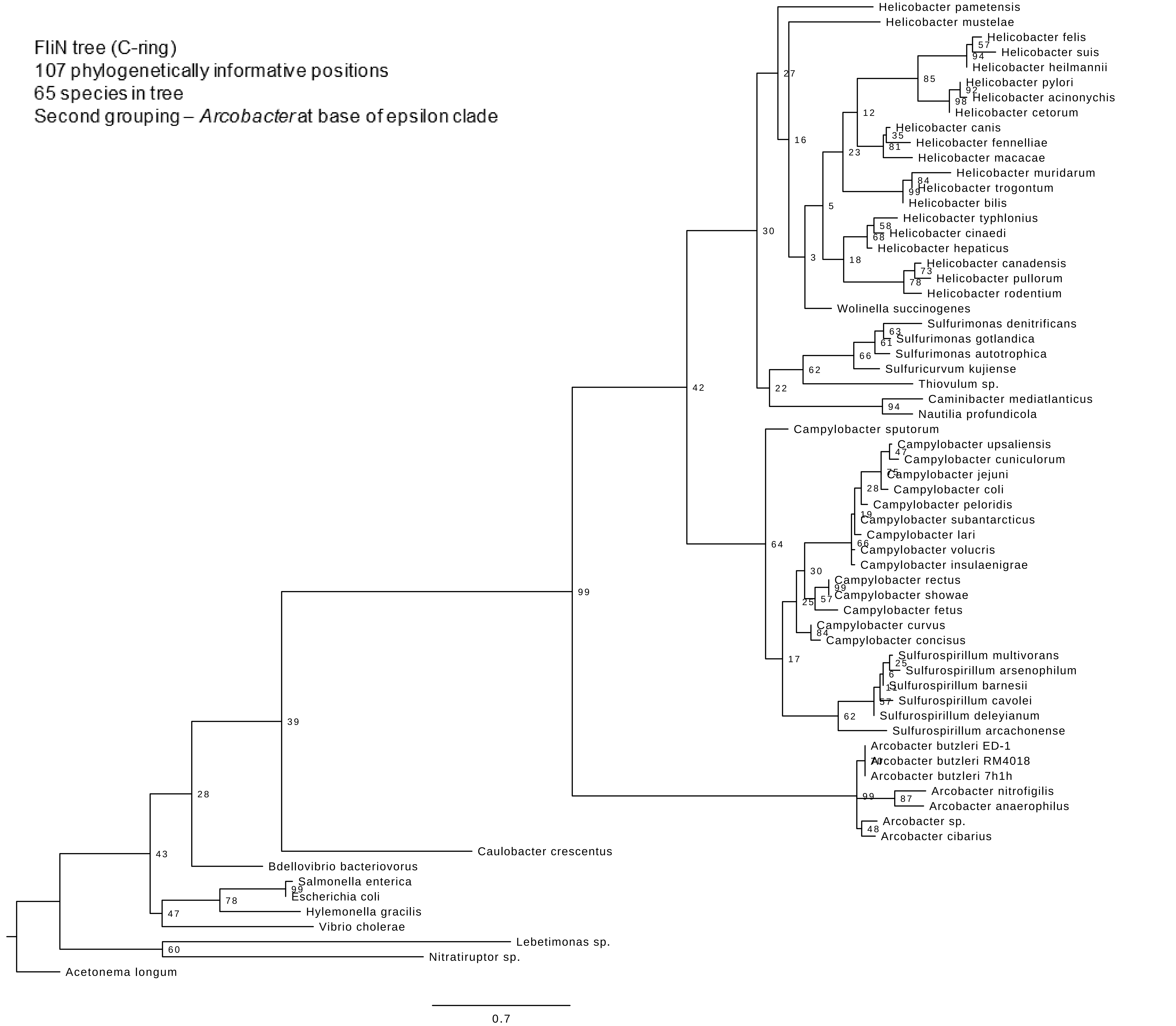

FliN tree (C-ring)
107 phylogenetically informative positions
65 species in tree
Second grouping – *Arcobacter* at base of epsilon clade
Helicobacter pametensis
Helicobacter mustelae
Helicobacter felis
Helicobacter suis
Helicobacter heilmannii
Helicobacter pylori
Helicobacter acinonychis
Helicobacter cetorum
Helicobacter canis
Helicobacter fennelliae
Helicobacter macacae
Helicobacter muridarum
Helicobacter trogontum
Helicobacter bilis
Helicobacter typhlonius
Helicobacter cinaedi
Helicobacter hepaticus
Helicobacter canadensis
Helicobacter pullorum
Helicobacter rodentium
Wolinella succinogenes
Sulfurimonas denitrificans
Sulfurimonas gotlandica
Sulfurimonas autotrophica
Sulfuricurvum kujiense
Thiovulum sp.
Caminibacter mediatlanticus
Nautilia profundicola
Campylobacter sputorum
Campylobacter upsaliensis
Campylobacter cuniculorum
Campylobacter jejuni
Campylobacter coli
Campylobacter peloridis
Campylobacter subantarcticus
Campylobacter lari
Campylobacter volucris
Campylobacter insulaenigrae
Campylobacter rectus
Campylobacter showae
Campylobacter fetus
Campylobacter curvus
Campylobacter concisus
Sulfurospirillum multivorans
Sulfurospirillum arsenophilum
Sulfurospirillum barnesii
Sulfurospirillum cavolei
Sulfurospirillum deleyianum
Sulfurospirillum arcachonense
Arcobacter butzleri ED-1
Arcobacter butzleri RM4018
Arcobacter butzleri 7h1h
Arcobacter nitrofigilis
Arcobacter anaerophilus
Arcobacter sp.
Arcobacter cibarius
Caulobacter crescentus
Bdellovibrio bacteriovorus
Salmonella enterica
Escherichia coli
Hylemonella gracilis
Vibrio cholerae
Lebetimonas sp.
Nitratiruptor sp.
Acetonema longum
0.7

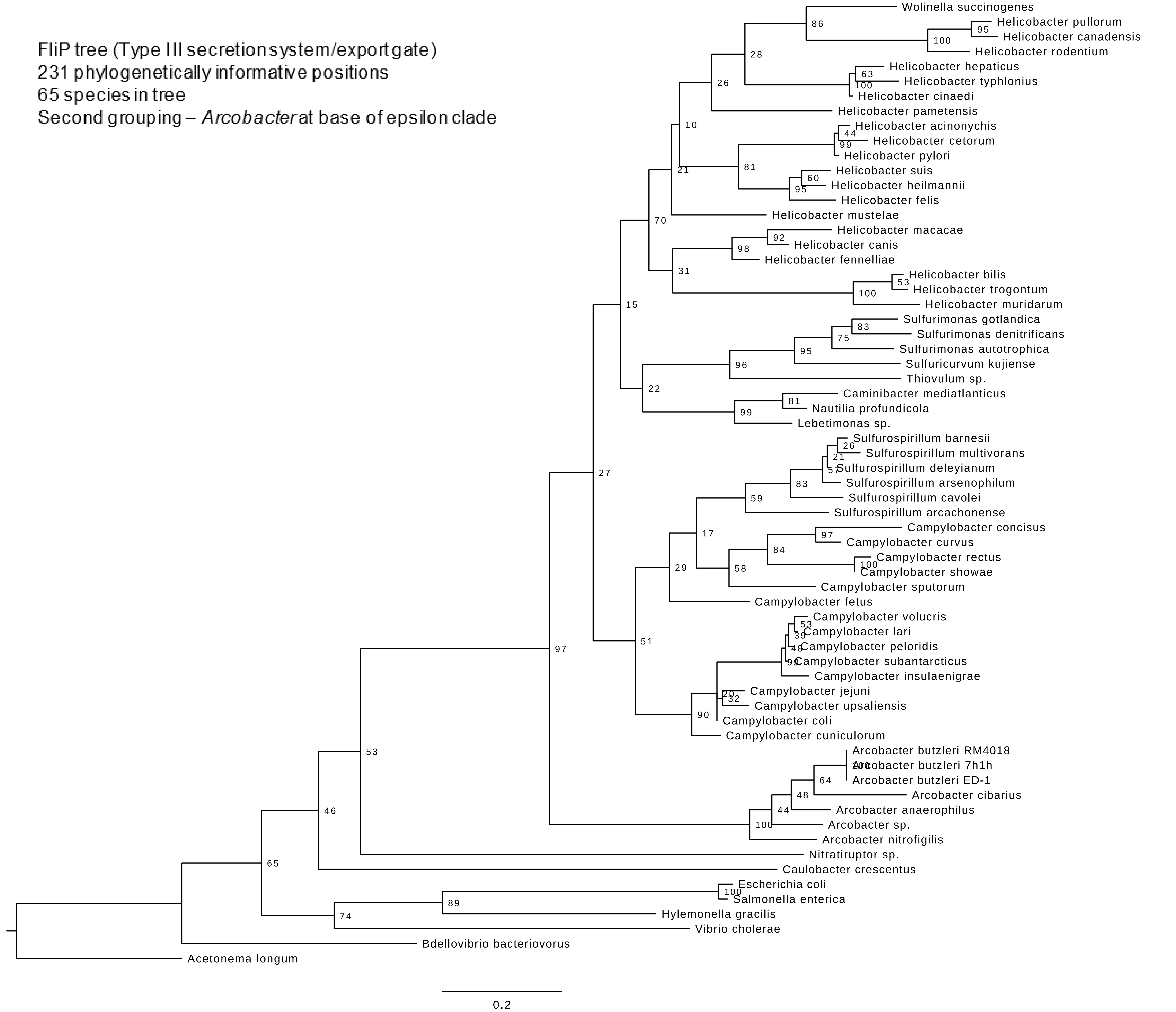
FliP tree (Type III secretion system/export gate)
231 phylogenetically informative positions
65 species in tree
Second grouping – Arcobacter at base of epsilon clade
Wolinella succinogenes
Helicobacter pullorum
Helicobacter canadensis
Helicobacter rodentium
Helicobacter hepaticus
Helicobacter typhlonius
Helicobacter cinaedi
Helicobacter pametensis
Helicobacter acinonychis
Helicobacter cetorum
Helicobacter pylori
Helicobacter suis
Helicobacter heilmannii
Helicobacter felis
Helicobacter mustelae
Helicobacter macacae
Helicobacter canis
Helicobacter fennelliae
Helicobacter bilis
Helicobacter trogontum
Helicobacter muridarum
Sulfurimonas gotlandica
Sulfurimonas denitrificans
Sulfurimonas autotrophica
Sulfuricurvum kujiense
Thiovulum sp.
Caminibacter mediatlanticus
Nautilia profundicola
Lebetimonas sp.
Sulfurospirillum barnesii
Sulfurospirillum multivorans
Sulfurospirillum deleyianum
Sulfurospirillum arsenophilum
Sulfurospirillum cavolei
Sulfurospirillum arcachonense
Campylobacter concisus
Campylobacter curvus
Campylobacter rectus
Campylobacter showae
Campylobacter sputorum
Campylobacter fetus
Campylobacter volucris
Campylobacter lari
Campylobacter peloridis
Campylobacter subantarcticus
Campylobacter insulaenigrae
Campylobacter jejuni
Campylobacter upsaliensis
Campylobacter coli
Campylobacter cuniculorum
Arcobacter butzleri RM4018
Arcobacter butzleri 7h1h
Arcobacter butzleri ED-1
Arcobacter cibarius
Arcobacter anaerophilus
Arcobacter sp.
Arcobacter nitrofigilis
Nitratiruptor sp.
Caulobacter crescentus
Escherichia coli
Salmonella enterica
Hylemonella gracilis
Vibrio cholerae
Bdellovibrio bacteriovorus
Acetonema longum
0.2

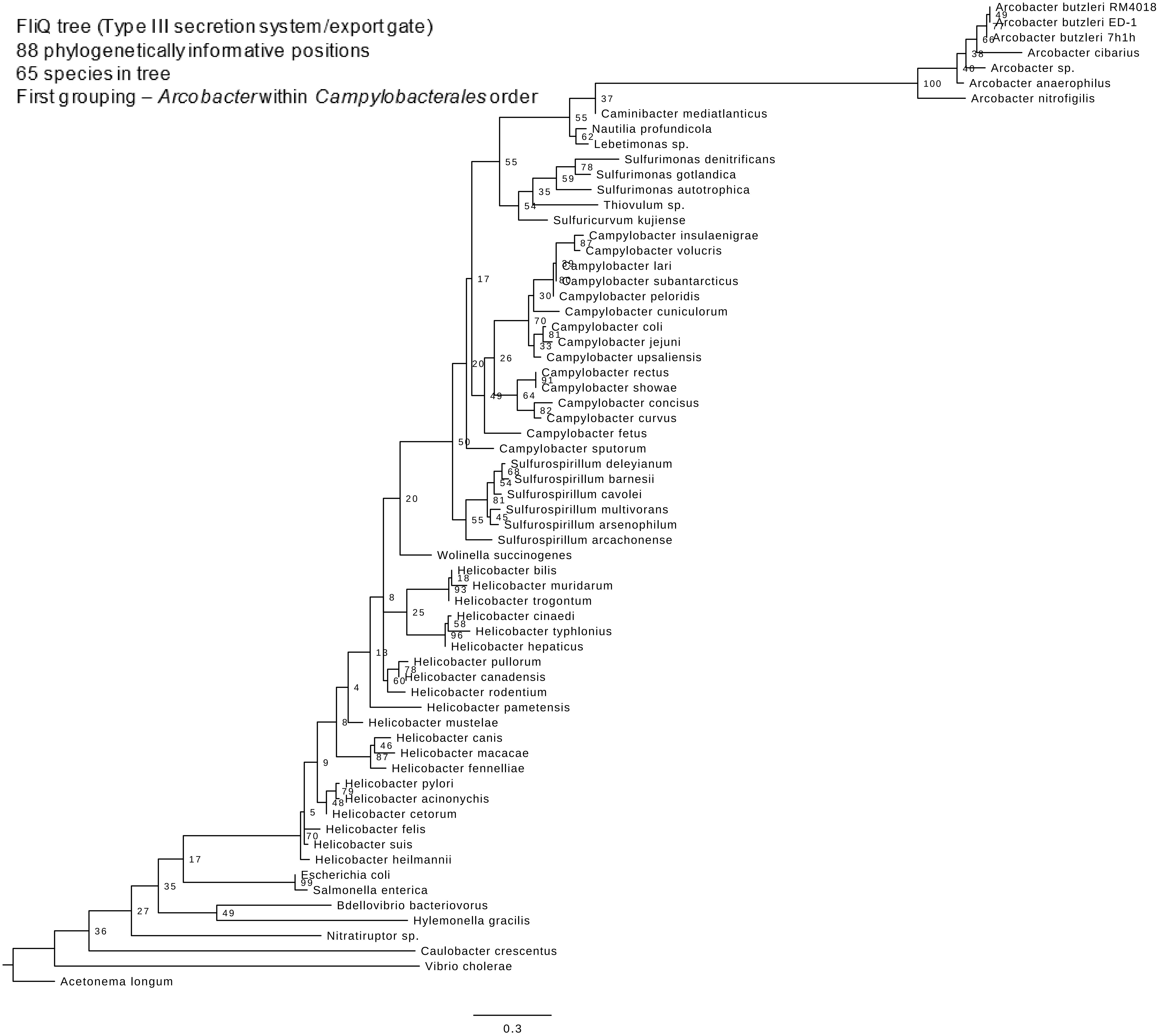

FliQ tree (Type III secretion system/export gate)
88 phylogenetically informative positions
65 species in tree
First grouping – Arcobacter within Campylobacterales order
Arcobacter butzleri RM4018
Arcobacter butzleri ED-1
Arcobacter butzleri 7h1h
Arcobacter cibarius
Arcobacter sp.
Arcobacter anaerophilus
Arcobacter nitrofigilis
Caminibacter mediatlanticus
Nautilia profundicola
Lebetimonas sp.
Sulfurimonas denitrificans
Sulfurimonas gotlandica
Sulfurimonas autotrophica
Thiovulum sp.
Sulfuricurvum kujiense
Campylobacter insulaenigrae
Campylobacter volucris
Campylobacter lari
Campylobacter subantarcticus
Campylobacter peloridis
Campylobacter cuniculorum
Campylobacter coli
Campylobacter jejuni
Campylobacter upsaliensis
Campylobacter rectus
Campylobacter showae
Campylobacter concisus
Campylobacter curvus
Campylobacter fetus
Campylobacter sputorum
Sulfurospirillum deleyianum
Sulfurospirillum barnesii
Sulfurospirillum cavolei
Sulfurospirillum multivorans
Sulfurospirillum arsenophilum
Sulfurospirillum arcachonense
Wolinella succinogenes
Helicobacter bilis
Helicobacter muridarum
Helicobacter trogontum
Helicobacter cinaedi
Helicobacter typhlonius
Helicobacter hepaticus
Helicobacter pullorum
Helicobacter canadensis
Helicobacter rodentium
Helicobacter pametensis
Helicobacter mustelae
Helicobacter canis
Helicobacter macacae
Helicobacter fennelliae
Helicobacter pylori
Helicobacter acinonychis
Helicobacter cetorum
Helicobacter felis
Helicobacter suis
Helicobacter heilmannii
Escherichia coli
Salmonella enterica
Bdellovibrio bacteriovorus
Hylemonella gracilis
Nitratiruptor sp.
Caulobacter crescentus
Vibrio cholerae
Acetonema longum
0.3

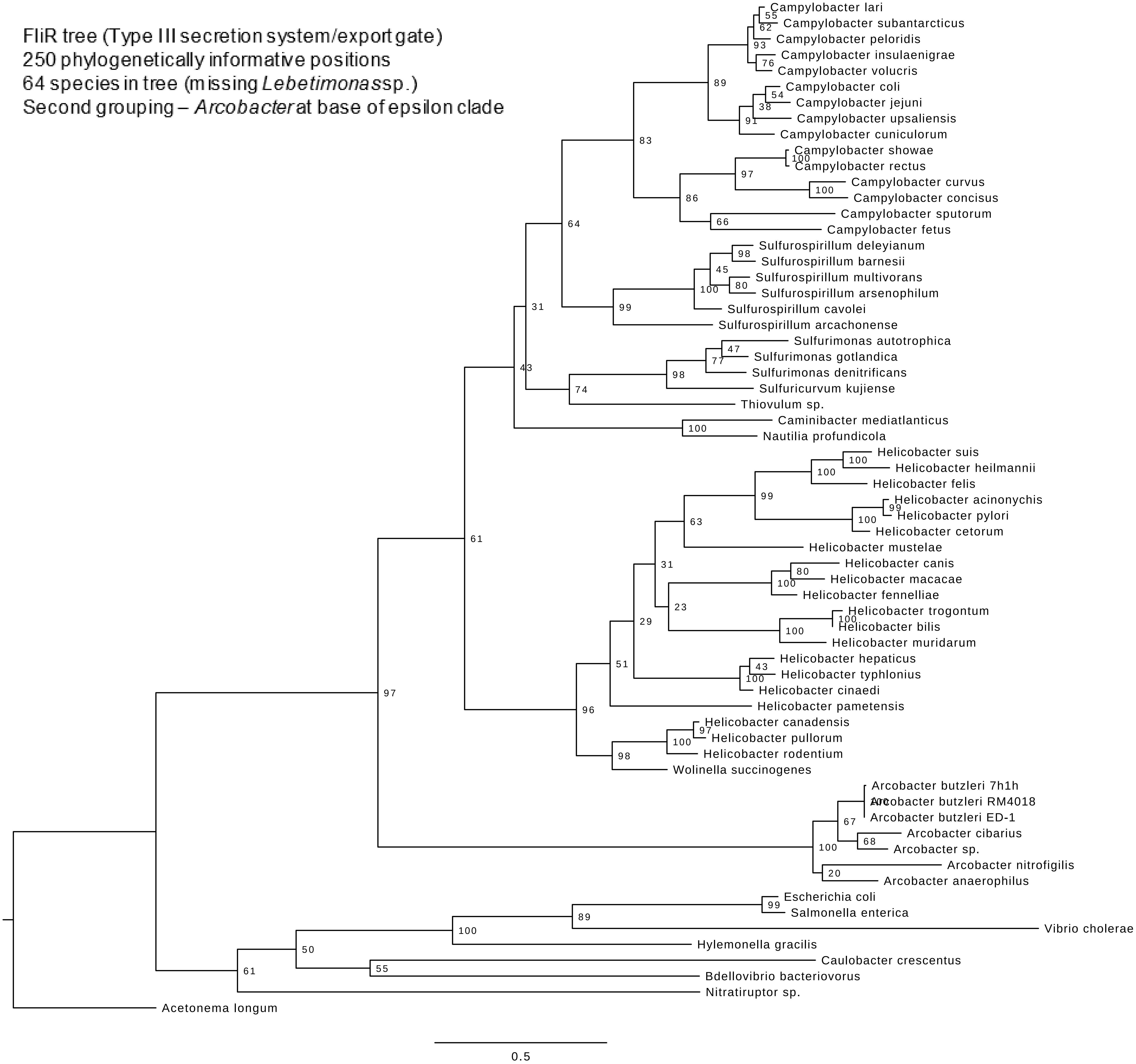
FliR tree (Type III secretion system/export gate)
250 phylogenetically informative positions
64 species in tree (missing *Lebetimonas* sp.)
Second grouping – *Arcobacter* at base of epsilon clade
Campylobacter lari
Campylobacter subantarcticus
Campylobacter peloridis
Campylobacter insulaenigrae
Campylobacter volucris
Campylobacter coli
Campylobacter jejuni
Campylobacter upsaliensis
Campylobacter cuniculorum
Campylobacter showae
Campylobacter rectus
Campylobacter curvus
Campylobacter concisus
Campylobacter sputorum
Campylobacter fetus
Sulfurospirillum deleyianum
Sulfurospirillum barnesii
Sulfurospirillum multivorans
Sulfurospirillum arsenophilum
Sulfurospirillum cavolei
Sulfurospirillum arcachonense
Sulfurimonas autotrophica
Sulfurimonas gotlandica
Sulfurimonas denitrificans
Sulfuricurvum kujiense
Thiovulum sp.
Caminibacter mediatlanticus
Nautilia profundicola
Helicobacter suis
Helicobacter heilmannii
Helicobacter felis
Helicobacter acinonychis
Helicobacter pylori
Helicobacter cetorum
Helicobacter mustelae
Helicobacter canis
Helicobacter macacae
Helicobacter fennelliae
Helicobacter trogontum
Helicobacter bilis
Helicobacter muridarum
Helicobacter hepaticus
Helicobacter typhlonius
Helicobacter cinaedi
Helicobacter pametensis
Helicobacter canadensis
Helicobacter pullorum
Helicobacter rodentium
Wolinella succinogenes
Arcobacter butzleri 7h1h
Arcobacter butzleri RM4018
Arcobacter butzleri ED-1
Arcobacter cibarius
Arcobacter sp.
Arcobacter nitrofigilis
Arcobacter anaerophilus
Escherichia coli
Salmonella enterica
Vibrio cholerae
Hylemonella gracilis
Caulobacter crescentus
Bdellovibrio bacteriovorus
Nitratiruptor sp.
Acetonema longum
0.5

Supplemental Information

"Evolution of higher torque in *Campylobacter*-type bacterial flagellar motors"

Bonnie Chaban[1†], Izaak Coleman[1], Morgan Beeby[1]*

[1]Department of Life Sciences, Imperial College of London, London, SW7 2AZ, UK

*corresponding author: MB: mbeeby@imperial.ac.uk, phone +44 (0) 20 7594 5251

[†]Current address: Faculty of Science, Health, Education and Engineering, University of the Sunshine Coast, 90 Sippy Downs Drive, Sippy Downs, 4556, QLD, Australia

Supplementary Figure S4: FSC curves of subtomogram averages, and side- and top-view slices through unsymmetrized and symmetrized structures. (Top to bottom): *Wolinella succinogenes*, *Arcobacter butzleri*, and *Bdellovibrio bacteriovorus*. FSC curves were determined using PEET's inbuilt *calcFSC* and *plotFSC* programs. Resolutions in Angstroms at a 0.5 threshold are 98 for *Wolinella succinogenes*, 82 for *Arcobacter butzleri*, and 80 for *Bdellovibrio bacteriovorus*.

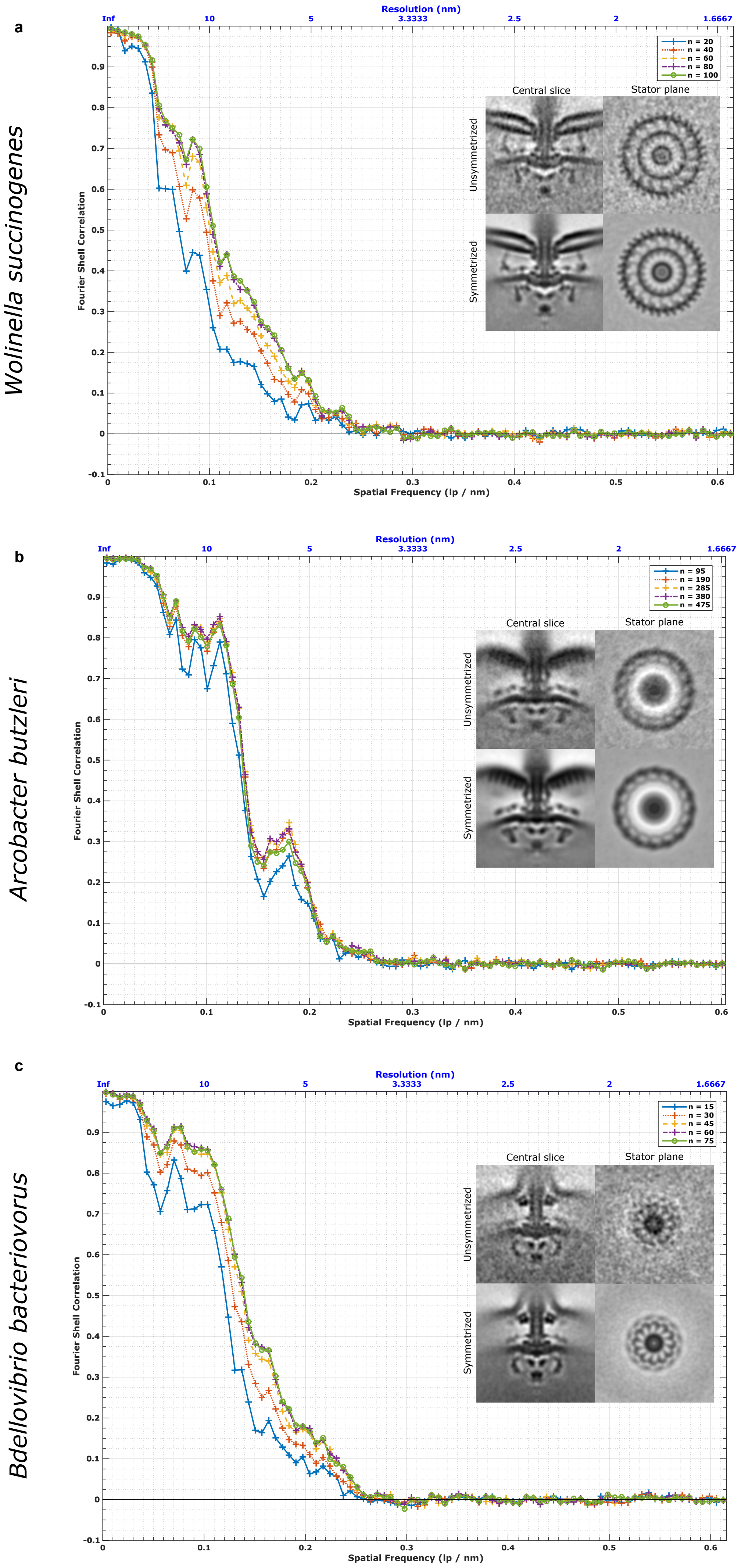

a
Wolinella succinogenes
Resolution (nm)
Inf
10
5
3.3333
2.5
2
1.6667
n = 20
n = 40
n = 60
n = 80
n = 100
Central slice
Stator plane
Unsymmetrized
Symmetrized
Fourier Shell Correlation
Spatial Frequency (lp / nm)
b
Arcobacter butzleri
Resolution (nm)
n = 95
n = 190
n = 285
n = 380
n = 475
Central slice
Stator plane
Unsymmetrized
Symmetrized
Fourier Shell Correlation
Spatial Frequency (lp / nm)
c
Bdellovibrio bacteriovorus
Resolution (nm)
n = 15
n = 30
n = 45
n = 60
n = 75
Central slice
Stator plane
Unsymmetrized
Symmetrized
Fourier Shell Correlation
Spatial Frequency (lp / nm)
